# Prediction of motion induced magnetic fields for human brain MRI at 3 T

**DOI:** 10.1007/s10334-023-01076-0

**Published:** 2023-03-25

**Authors:** Jiazheng Zhou, Gisela E. Hagberg, Ali Aghaeifar, Jonas Bause, Maxim Zaitsev, Klaus Scheffler

**Affiliations:** 1 Max Planck Institute for Biological Cybernetics, High-Field Magnetic Resonance Center, Max-Planck-Ring 11, 72076 Tübingen, Germany; 2 IMPRS for Cognitive and Systems Neuroscience, University of Tübingen, Tübingen, Germany; 3 Department of Biomedical Magnetic Resonance, University Hospital Tübingen, Tübingen, Germany; 4 Department of Radiology, Medical Physics, Faculty of Medicine, Medical Center University of Freiburg, University of Freiburg, Freiburg, Germany

**Keywords:** B_0_ homogeneity, UTE, Susceptibility model, Head motion

## Abstract

**Objective:**

Maps of B_0_ field inhomogeneities are often used to improve MRI image quality, even in a retrospective fashion. These field inhomogeneities depend on the exact head position within the static field but acquiring field maps (FM) at every position is time consuming. Here we propose a forward simulation strategy to obtain B_0_ predictions at different head-positions.

**Methods:**

FM were predicted by combining (1) a multi-class tissue model for estimation of tissue-induced fields, (2) a linear k-space model for capturing gradient imperfections, (3) a dipole estimation for quantifying lower-body perturbing fields (4) and a position-dependent tissue mask to model FM alterations caused by large motion effects. The performance of the combined simulation strategy was compared with an approach based on a rigid body transformation of the FM measured in the reference position to the new position.

**Results:**

The transformed FM provided inconsistent results for large head movements (> 5° rotation, approximately), while the simulation strategy had a superior prediction accuracy for all positions. The simulated FM was used to optimize B_0_ shims with up to 22.2% improvement with respect to the transformed FM approach.

**Conclusion:**

The proposed simulation strategy is able to predict movement-induced B_0_ field inhomogeneities yielding more precise estimates of the ground truth field homogeneity than the transformed FM.

**Supplementary Information:**

The online version contains supplementary material available at 10.1007/s10334-023-01076-0.

## Introduction

Spatial encoding and signal acquisition in Magnetic resonance imaging (MRI) rely on a homogenous static magnetic field (B_0_). Spatially varying magnetic susceptibilities within samples can perturb the static magnetic field. These perturbations are prominent at the boundaries of tissues with distinct susceptibilities. In particular, significant perturbative effects originating from anatomic air cavities and biological tissue borders can lead to local frequency deviations and reduce the quality of magnetic resonance measurements. For example, B_0_ field inhomogeneity causes signal voids and geometric distortion in gradient recalled echo-echo planar images (GRE-EPI) [1]. Signal loss due to intravoxel dephasing can be partly recovered through the use of acquisition-based methods, such as Z shimming [2] or hybrid RF pulse design [3]. The geometric distortion artifact could be corrected or substantially reduced in images through post-processing [4] or B_0_ shimming [5], provided that knowledge of the field distribution is available.

Long-lasting experiments (e.g., fMRI) are known to be prone to subject head movements. According to recent assessments, involuntary subject motion is commonly observed even in typical fMRI experiments of young, motivated volunteers, with approximately 1 to 2 mm translation, and rotations of approximately 1 to 2 degrees [6]. In the case of patients, elderly and children, however, substantially more obtrusive motion is oft observed. The head motion alters the position and orientation of the susceptibility interfaces in relation to the static B_0_ field and accordingly alters the inhomogeneity distribution. Therefore, the measured field map (FM) acquired only once may not be valid for the correction of geometric distortions for the entire fMRI session. A typical whole-brain field map acquisition with dual-echo GRE takes 1–3 min depending on the field-of-view (FOV), resolution, and acceleration provided by parallel imaging. Due to time constraints, it may be impractical to repeat it several times for each and every head position. As an alternative, field maps can be calculated from phase offsets of two EPI measurements [7, 8]. However, this requires a sequence modification to implement jittered echo time [9]. Additionally, methods like 3D EPI navigator [10] and FID navigator [11] can be implemented to obtain a rapid estimation of the field map. Two major disadvantages of these techniques include the difference in echo time between the navigator and read-out as well as subject motion during the acquisition of the navigators or between EPI echo trains.

In principle, the magnetic field map could be obtained from analytic magnetostatic equations [12], if all susceptibility sources, including their shape, and orientation with respect to the external field were known. However, analytical solutions are only practical for simplified geometries (like spheres or cylinders), making this approach very difficult to be used with complex structures. Alternatively, rapid macroscopic dipole approximation methods [13, 14] give a numerically approximated solution of susceptibility induced B_0_ field inhomogeneities. This allows the estimation of B_0_ field inhomogeneities for arbitrary sample structures and significantly reduces computational time. The mentioned computational approach requires an accurate construction of susceptibility models specific to each sample. When imaging a phantom, such models can be constructed based on the design data and material properties [15]. In the human head, the primary susceptibility components are the brain tissue, bone, and air. Therefore, initial studies were based on the fusion of computed tomography (CT) and MRI images to make such a head model [16, 17]. Although the susceptibility model is helpful, it is not subject-specific and depends on the co-registration process to accurately localize the sinuses in MR images. One possibility to observe and separate air/bone boundaries using MR images only, is by utilizing an ultra-short echo time (UTE) sequence which is suitable for the detection of signals from tissues with very short T2 components (e.g., bone) with nominal TEs <  < 1 ms. A similar approach using a dual-echo, short TE, 3D GRE sequence for air-tissue boundary segmentation has provided promising results [18] for background field removal applications such as susceptibility-weighted imaging (SWI) [18] and quantitative susceptibility mapping (QSM) [19]. Such simplified models may not reflect the full complexity of the B_0_ field generated by the human head.

Here, we propose a field map simulation approach to predict the B_0_ field at different head positions. It encompasses both global and more local effects based on a susceptibility model built from UTE scans. We systematically include four components for field map prediction: (1) a multi-class tissue model for estimation of tissue-induced fields, (2) a linear k-space model for capturing gradient imperfections, (3) a dipole estimation for quantifying lower-body perturbing fields (4) and a position-dependent tissue mask to model FM alterations caused by large motion effects. For validation, we compared the performance of our simulated FMs with ground-truth measured FMs acquired at different head positions as well as with results obtained with a method that simply does a rigid-body, spatial transformation of the FM measured at the reference position [20]. One of the possible uses of our approach in the future is real-time shimming. As a demonstration of the utility of our approach, we therefore used the simulated FM to obtain novel B_0_ shim currents and compared the field homogeneity attained with that obtained from a standard shimming approach.

## Method

**Fig. 1:**
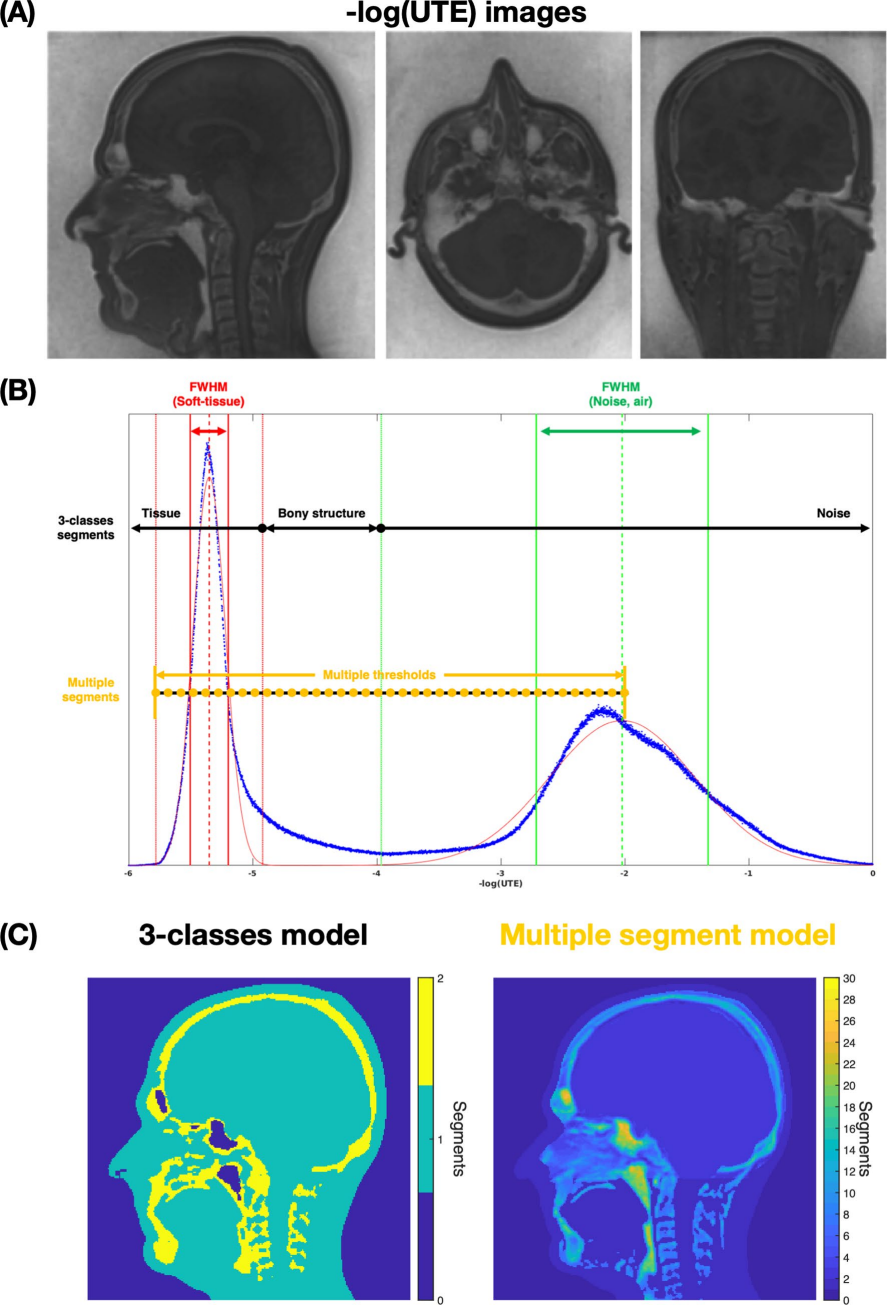
The threshold-based air, bone, and soft tissue segmentation of inverting the logarithmically scaled UTE dataset. **A** Three orthogonal views of the inverted logarithmically scaled UTE image, with full range [− 7,0]. **B** The image histogram with two distinctive peaks as soft-tissue (left) and noise (right) signals. Gaussian-fitting results with center peak (dashed line) and full-width-half-maximum (FWHM) information (dashed lines) for soft-tissue (red) and noise (green). The 3-class segment model is based on the bone signal threshold [Center (soft) + 1.4*FWHM (soft), Center (noise)-1.4*FWHM (noise)], which is positioned in between the two peaks, leading more toward soft tissue. The multiple segment model is defined as a linear interval between [Center (soft)-1.4*FWHM (soft): 0.1: Center (noise)]. **C** Middle sagittal slices of 3-classes segment model and multiple segment model

**Fig. 2:**
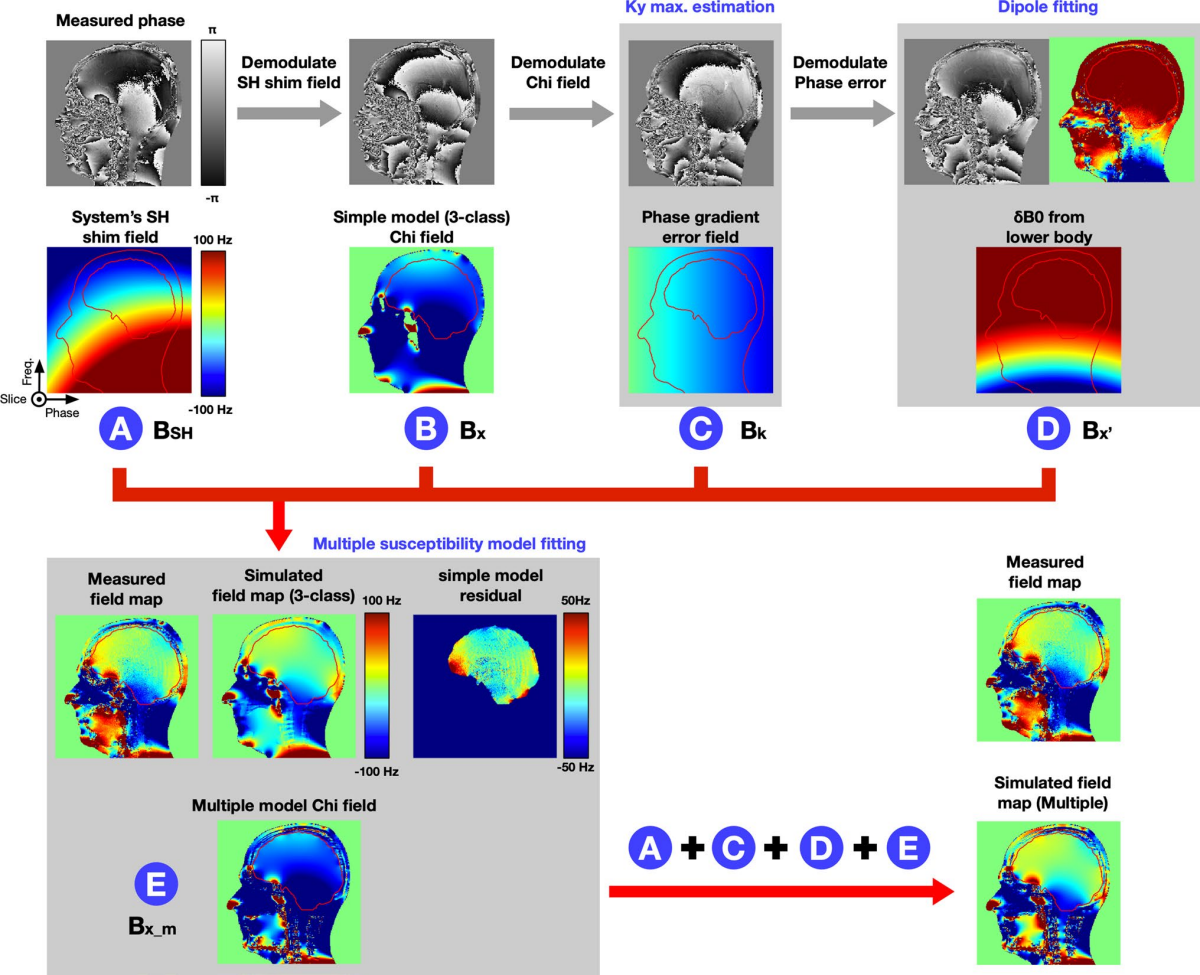
Schematic of simulated field map. The simulation field map is based on a 5 steps magnetic field estimation: **A** the system SH shim field is calculated from the SH shim coefficients; **B** the forward approximated sample induced *B*_χ_ field; **C** Linear phase errors by the imaging gradients; **D** Dipole approximation of the *B*_χ’_ field from the lower body part; **E** Susceptibility model fitting to reduce the simulation residual error with the *B*_χ_m_ field

**Fig. 3:**
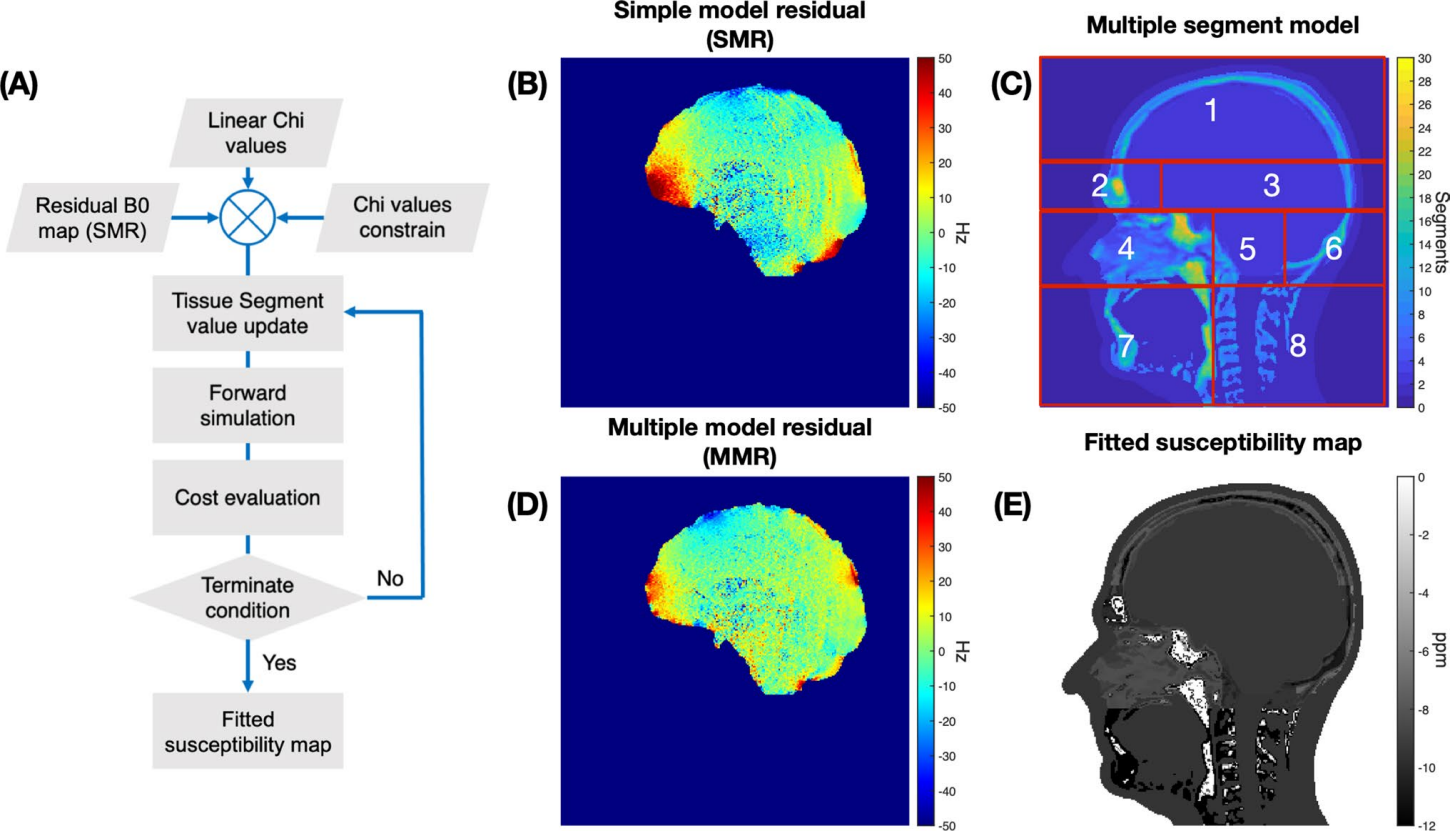
**A** Schematic of the iterative fitting algorithm. **B** The simple model residual field map, SMR, when using a 3-class susceptibility map. **C** The multiple segment model with additional spatial anatomical constrains. **D** The multiple model residual field map, MMR, when using a fitted susceptibility map **E** The fitted susceptibility map for one volunteer

**Fig. 4:**
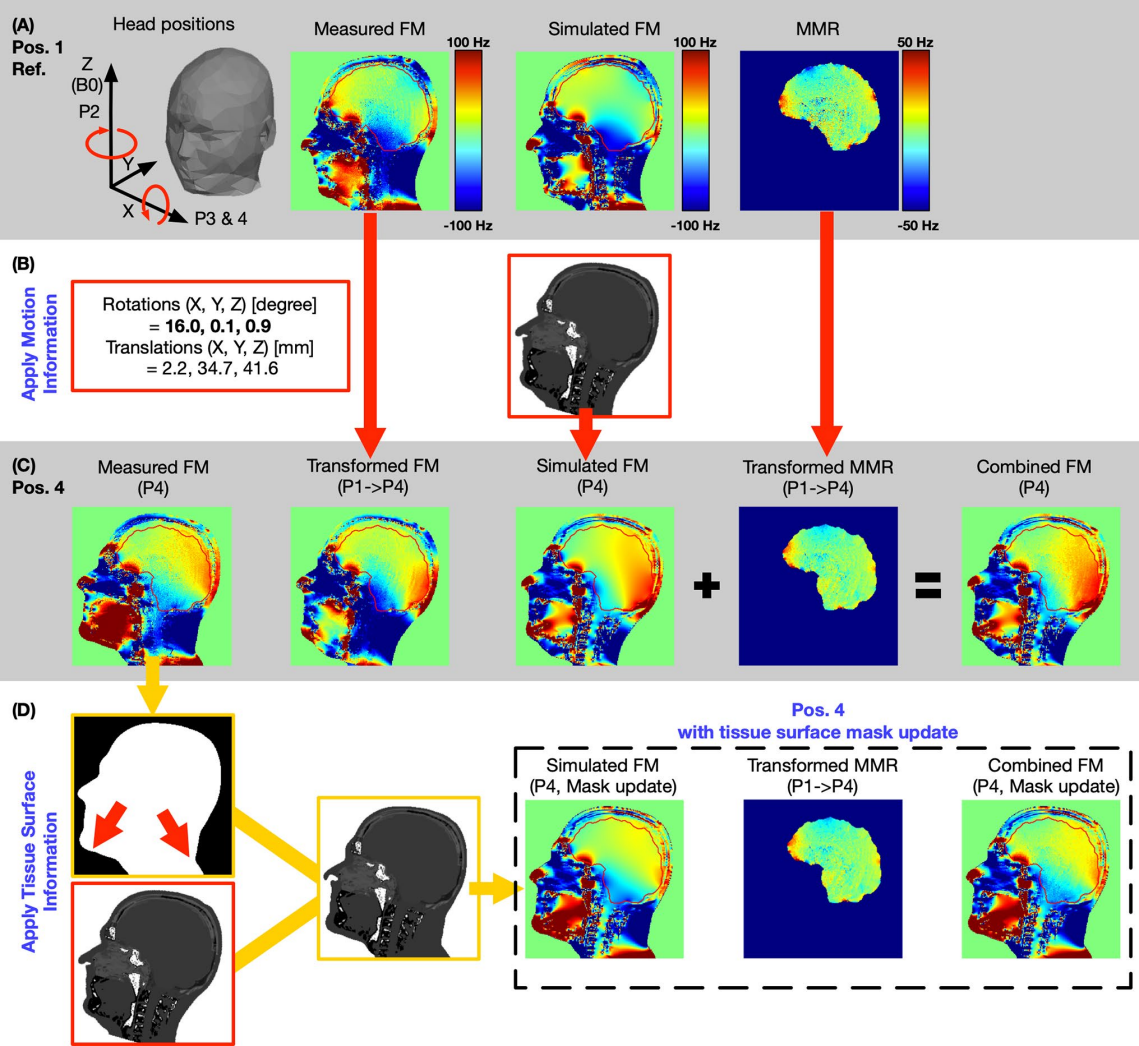
**A**, **B**, **C** illustrates the calculation of transformed FM, simulated FM, and combined FM from a reference position (Position 1) to a new head position (Position 4). The transform FM utilizes the measured FM in reference position with a simple rigid-body transform operation. The absolute rotation and translation values have been marked in red squares. The simulated FM is calculated from the forward B_0_ approximation, with a rigid-body transformed susceptibility map. The combined FM adds a rigid-body transform MMR field map from Position 1 into the simulated FM at a new head position. **C** and **D**, show the process of susceptibility map mask adjustment when the large head rotation on X-axis causes subject tissue boundary changes. The updated head-mask is calculated from the measured field map at Position 4 and applied to the rigid body transformed susceptibility map. For large head movement, the simulated FM and combined FM were recalculated using the head-mask updated susceptibility map (dash square). The red arrow indicates the mask displacement

Our approach can be subdivided into three parts: (1) acquisition of MRI data and tissue segmentation (Fig. 1), (2) B0 map simulation (Fig. 2, 3); Field map prediction for different head positions (motion-prediction) (Fig. 4).

### MRI acquisition and tissue segmentation

All measurements were performed on a Siemens Prisma Fit 3 Tesla scanner (Siemens Healthineers, Erlangen, Germany). Scans were performed by using the scanner body coil for RF transmission and the 64-channel head array for signal reception. Four healthy volunteers (3 male and 1 female) were recruited and provided a written informed consent after a full explanation of the protocol. The study was conducted in accordance with the University Hospital of Tuebingen Ethics Review Board.

Experiments involved acquisition of field map and structural images from each volunteer and using these to form head models and obtain field maps at four head positions. In the first head position, 2^nd^ order spherical harmonics (SH) shimming was performed for the whole head and neck region and frequency adjustment was performed followed by a series of image acquisitions including: (1) Dual‐echo 3D GRE sequence to measure reference field maps for the subsequent calculations. Specifically, the first phase encoding direction (PE) of the 3D GRE sequence is along the Y-axis of the scanner (the Anterior–Posterior axis of the head), the second phase encoding (partition encoding) is applied along the X-axis of the scanner (Right–Left of the head), and the read-out direction is along the Z-axis of the scanner (Head—Feet), (TE_1/2_ = 2.68/7.49 ms, TR = 11 ms, FA = 12°, FOV = 256*256*192 mm^3^, voxel size = 1 mm^3^ isotropic, GRAPPA factor = 2, monopolar readout gradients and “whisper” gradient mode, which reduces the slew rate, were used to minimize possible eddy currents); (2) 3D stack-of-spiral UTE sequence for imaging short T2 anatomical components (e.g., bone), which has the same FOV and FOV location as the 3D GRE sequence (Siemens WIP 992D, TE = 0.05 ms, TR = 8 ms, FA = 1°, FOV = 256*256*192 mm^3^, voxel size = 1 mm^3^ isotropic). Next, the volunteers were asked to rotate their heads about the Z-axis (Position 2) and the X-axis both clockwise and anti-clockwise (Position 3 look down, Position 4 lookup). The dual-echo 3D GRE sequence was repeated to measure reference FM at each head position: Position 2, Position 3, and Position 4. Subject-specific SH shim parameters and the Tune-up parameters were recorded and subsequently used for the simulations. The SH shim remained the same during the entire experiment, in order to imitate the SH shimming condition where the volunteer moves and takes on different head positions during an experimental session.

Tissue compartments, including bone, soft tissue, and cavities were segmented from the proton density weighted UTE image using a threshold-based segmentation method [21]. The UTE image was first bias-corrected for spatial intensity variations using an N4-bias filter [22] (Fig. 1A). Then an image histogram was generated after inverting the logarithmically scaled UTE dataset (-log (UTE)). The soft tissue and air appeared as two distinct peaks where the peak with small values corresponding to soft tissue and large values to air-filled cavities (Fig. 1B). The bone signals are spread in-between these peaks, trending more toward the soft tissue peak. Two different tissue segment models were developed (Fig. 1C). The first model is a 3-class model where the -log (UTE) image was used to segment the head into air, bone, and soft tissue using an empirically chosen bone threshold derived from a Gaussian fit of the soft-tissue and noise peaks, ranging from [Center (soft) + 1.4*FWHM (soft), Center (noise)-1.4*FWHM (noise)] (Fig. 1B). This 3-class model was later used for the forward calculation of the susceptibility induced field as described in Sect. 2.2-compartment B. However, several voxels in the nasal area and ear canals often yielded misclassification, since these cavities often contain complex structures comprising soft tissue, mucus, air, and cartilage, which usually results in partial volume effects. To account for that, a brain mask was first generated with the brain extraction tool (BET) [23] in the FMRIB Software Library (FSL) [24]. Voxels outside this mask were subdivided into multiple segments based on their image intensity in the -log (UTE) image. The linear interval between [Center (soft)-1.4*FWHM (soft): 0.1: Center (noise)] (Fig. 1B) was subdivided into bins with a size of 0.1, to cover nearly all intermediary tissue classes and cavities. Specific susceptibility values for each tissue class (tissue bin) within the multiple segments model were identified from an iterative algorithm (as described in Sect. 2.2-compartment E). These settings and procedures were reproducible across all participating volunteers. The segmentation, simulation process, and modeling were developed in MATLAB (MathWorks Inc., Natick, USA).

## Field map simulation

The deviation (ΔB_0_) from a homogeneous magnetic field can be approximated by a superposition of different components. Once a sample is positioned in the bore, the sample (e.g., the human head) results in field inhomogeneities *B*_χ_ (on the order of a few ppm of the static field) while the static SH shim field generated by the scanner *B*_SH_ will be optimized to counteract the magnetic field inhomogeneities caused by the sample. Additional components are the field generated by imperfect gradients system that can include other potential errors in the phase encoding direction *B*_k_, during the 3D GRE field mapping, and the perturbing field *B*_χ’_ originating from tissue susceptibility sources (e.g., lungs) located outside the FOV.

Thus, the simulated field map ΔB_0_ can be described as Eq. 1 (Fig. 2):

1 $$\Delta B_{0} = B_{SH} + B_{\chi } + B_{k} + B_{\chi }$$

Two components were excluded since our focus is on the human brain. The first component is the static magnetic field B_0_ which is commonly treated as a constant. However, in scans with FOV larger than those used for the human head, the static magnetic field cannot be assumed to be constant across the entire imaging volume. Secondly, chemical shift effects *B*_σ_ from lipids, which play a minor role in predicting the field in brain tissue (as shown in the supplementary figure S1). Therefore, in our simulation, we did not consider any chemical shift effects inside the brain. Nevertheless, for other body parts (e.g., liver), both chemical shift and sample susceptibility effects are linearly equally dependent on the main static field strength and need to be considered. Conventional phase contrast cannot distinguish between both sources; therefore, chemical shifts can falsify measurements in MRI when not considered properly. Figure 2A reports the results of SH shim field (*B*_SH_) simulation.

The SH shim field from the scanner could be either measured on a spherical phantom or simulated from the vendor-provided SH shim coefficients. In an experiment with large head motion induced, the position of the subject’s head may fall outside a volume defined by the phantom dimensions. Thus, we opted to simulate the SH shim field (up to 2nd order SH) within the imaging FOV instead of measuring it (Fig. 2A). Figure 2B reports the results of sample susceptibility induced field (*B*_χ_) using forward calculation.

To rapidly compute magnetic field inhomogeneities over arbitrary sample geometries, we used the Fourier dipole approximation method derived by Marques and Bowtell [14] and Salomir [13] et al. This method only requires Fourier transforms over the input susceptibility distributions, with additional consideration of the Lorentz sphere correction term for the microscopic susceptibility correction from Maxwell’s equations, as described by Eq. 2.

2 $${B}_{\upchi }\left({\varvec{r}}\right)={FT}^{-1}\left\{{B}_{0}\left[\frac{1}{3}-\frac{{k}_{z}^{2}}{{k}_{x}^{2}+{k}_{y}^{2}+{k}_{z}^{2}}\right]\cdot \widetilde{\chi }\left({\varvec{k}}\right)\right\}$$

Here the $\widetilde{\chi }$ indicates the 3-dimensional Fourier transform of the susceptibility distribution and ***k*** indicates the k-space vector. Susceptibility is weighted by a k-space scaling factor (the terms in brackets), which represents the Lorentz sphere corrected dipole response of the system to an external field. The susceptibility-induced magnetic field in image-space is then given by the inverse Fourier transform of the term in curly brackets.

A simple susceptibility map was first determined by assigning typical tissue susceptibility values from the literature to the 3-class model [air = 0 ppm (reference), bone = − 11.4 ppm[19], soft tissue = − 9.6 ppm[25]] (Fig. 1C left). The three-class susceptibility map was used to rapidly calculate the contribution from sample susceptibility induced field inhomogeneities (*B*_χ_, Fig. 2B).

Figure 2C reports the results of estimation of phase error *B*_*k.*_

Although the dual-echo 3D GRE sequence protocol was optimized to minimize possible eddy current effects, we still observed some phase error contributions (Fig. 2C).

These errors were modeled by linear fitting of the maximal k_y_ shift of the complex signal in the k-space center for each echo time (*t*_*n*_) after demodulating the effects of SH shim and subject specific *B*_χ_ field from the measured dual-echo 3D GRE sequence (Eq. 3).

3 $${\Delta {\varvec{k}}}_{n}= -{\varvec{a}}{t}_{n}$$

where, the *Δk*_*n*_ is the k-space shift for echo time *t*_*n*_, and **a** is the slope. We set the linear fit intercept ***b*** to zero, since *Δk*_*0*_ = *t*_*0*_ = 0. Further discussion of the potential sources of this field contribution can be found in the supplementary document and in the Discussion Sect. 4.2.

Figure 2D reports the results of magnetic dipole fitting of *B*_χ’._

The forward calculation method requires a whole sample susceptibility model (e.g., the entire human body), which is typically not available if the sample susceptibility model is from a scan with limited FOV (e.g., human head or brain). But the ΔB_0_ field in the smaller FOV will also be impacted by a perturbing field *B*_χ’_ from sources located outside this FOV. Here we approximate the *B*_χ’_ field by a single magnetic dipole field, generated by a source located outside of the region of interest [26].

Equvation. 4 shows the perturbing field *B*_χ’_ at position ***r***, modelled as an axially oriented magnetic dipole. It is given as a function of four parameters, the dipole strength P and position ${{\varvec{r}}}_{{\varvec{d}}}={x}_{d}\widehat{{\varvec{x}}}+ {y}_{d}\widehat{{\varvec{y}}}+{z}_{d}\widehat{{\varvec{z}}}$, of the dipole.

4 $$B_{{\chi^{^{\prime}} }} \left( r \right) = \frac{P}{{\left| {r - r_{d} } \right|^{3} }}\left( {3\left( {\frac{{\left( {r - r_{d} } \right) \cdot \hat{z}}}{{\left| {r - r_{d} } \right|}}} \right)^{2} - 1} \right)$$

The *P* and ***r***_**d**_ were determined by minimizing a cost function (Eq. 5), which was defined as the root-mean-square error (RMSE) between the *B*_χ’_ and ${B}_{diff}= {B}_{measured}-{B}_{SH}-{B}_{\upchi }-{B}_{k}$, using the “fminsearch” function in MATLAB,

5 $$\cos t = \sqrt {\frac{1}{n}\mathop \sum \limits_{r = 1}^{n} \left( {B_{diff} \left( r \right) - B_{{\chi^{^{\prime}} }} \left( r \right) - B_{0\_shift} } \right)^{2} }$$

This method is valid since the field contributions from the other sources/factors have been mostly removed (*B*_*diff*_) or are significantly smaller, which leaves only a “linear” like perturbing field in the brain (see Fig. 2D). A constant B_0_shift_ factor (to make sure the simulated FM and measured FM have similar zero offsets) was estimated, together with the lower body induced *B*_χ’_.

Figure 2E reports the results of refined sample susceptibility induced field (*B*_χ_m_).

The simple (three-class) susceptibility map was used to rapidly calculate field contributions of *B*_k_, and *B*_χ’_ (Fig. 2C, D). Compared to the measured field map, the residual field obtained after demodulating the field contributions of *B*_χ_,* B*_SH_, *B*_k_, and *B*_χ’_ is relatively small (Fig. 2E), however it shows localized maxima close to air cavities. Thus, we hypothesized that this simple model residual field, SMR, mainly reflects incorrectly assigned tissue and cavity boundaries, and differences between the subject-specific susceptibility values and literature values when using a 3-class susceptibility map. Within the imaging FOV, the field contributions, such as *B*_SH_, *B*_k_, and *B*_χ’_ are independent of susceptibility sources within the brain. Therefore, we used an iterative nonlinear optimization algorithm to fit a more refined subject-specific susceptibility model.

Intuitively, by identifying the susceptibility values within each voxel a more detailed and subject specific *B*_χ_m_ field can be obtained. We use the “fmincon” function in MATLAB (Fig. 3A) to approximate the susceptibility values in a multi-class tissue model for all voxels outside the brain (Fig. 1C), by minimizing a cost function (Eq. 6) defined as the RMSE between the *B*_χ_m_ field and the field map difference at the reference position ${B}_{diff\_m}= {B}_{measured}-{B}_{SH}-{B}_{{\upchi }^{^{\prime}}}-{B}_{k}$

6 $$cost\_m=\sqrt{\frac{1}{n}\sum_{r=1}^{n}{({B}_{diff\_m}\left({\varvec{r}}\right)-{B}_{\upchi \_\mathrm{m}}\left({\varvec{r}}\right))}^{2}}$$

Susceptibility values within the same segmentation class could be different between different anatomical regions (e.g. the ear canals and the frontal sinuses), therefor the minimization is performed for eight volumes of interest (VOIs) that were defined to target brain regions with different types of air-tissue interfaces within the multiple segment model (supplementary figure S2): (1) top skull, (2) frontal sinus, (3) middle skull, (4) nasal cavities, (5) ear canals, (6) lower skull, (7) jaw and airway and 8) spine. The same tissue class within each different VOIs, could have different susceptibility values, and the susceptibility is determined only for undefined voxels located outside the brain mask. Additionally, to reduce the number of voxels that requires fitting, hence, to reduce the computational time, all voxels within the VOIs in the jaw and neck regions that could be assigned as brain, based on the BET brain mask, or muscle tissue, based on the -log (UTE) signal, were assigned the susceptibility values from the literature (Fig. 3E). For the initial starting point, we used a linear susceptibility interval of [− 12 ppm, 0 ppm], where the incremental step was calculated to match the segmented tissue classes in the multiple segment model. The higher -log (UTE) signal, the higher the susceptibility value. In the iterative steps, we use a lower and upper bound for the susceptibility values of [− 14 ppm, 1 ppm]. Further discussion of this method can be found in Discussions Sect. 4.3 and in the supplementary Figure S6, explaining the trade-off between computational time and level of detail of the multi-class model.

### Motion induced B_0_ estimation

Field maps were predicted for each head position and compared with the actual field map, measured in the new position (Fig. 4A–C). The predicted field maps were obtained in three different ways: (a) after a rigid-body transform of the field map measured in the reference position (transformed FM), (b) by a simulation strategy (Simulated FM), or by (c) a combined simulation strategy (Combined FM). The rigid-body transform parameters were obtained from an image-based retrospective correction method, FLIRT [27], in the FSL [24] package.

For the field-map transform strategy (Fig. 4A–C), the calculated field map at the new head position was obtained through a rigid-body transformation of the measured field map acquired at the reference position (Position 1). Note that the static SH shim field (*B*_*SH*_) was first subtracted from the measured field map at the reference position in order to approximate the subject-induced ΔB_0_ variations. The transform strategy is similar to a previously proposed template-based prediction method [20].

For the simulation strategy (Fig. 4A–C), the field map at each new head position was first approximated from Eq. 1, described in Sect. 2.2. At each new head position, we assumed that the SH shim field (*B*_*SH*_), phase error (*B*_*k*_), and *B*_χ_ from the lower body (*B*_χ’_) remained unchanged since the head movement won’t affect these factors. Starting from the *B*_χ_m_ at the reference position, the position-dependent susceptibility model was obtained by rigid-body transformation of the fitted susceptibility model at the reference position. Accordingly, the simulated FM approach is thus composed of components A, C, D and E as described in Sect. 2.2.

For the combined strategy (Fig. 4A–C), the multi-class model residual field map, MMR, was included by calculating the difference between the measured and simulated field map at the reference head position (Position 1), which first underwent a rigid-body transformation and was then added to the simulated field map at the new head position. Therefore, the combined FM approach is composed of components A, C, D, E and MMR. The combined strategy represents a simple derivative step of the simulation strategy by including terms that arise through error propagation and/or through sources not otherwise accounted for (e.g., material with different susceptibility trapped in the outer mucosa or in the ear canal). We included the transformed MMR in the simulation field map in order to mitigate the difference between the simulated field map and the measured field map at each new head position. The MMR thus reflects residual local field sources that we could not fully model as well as noise in the phase image.

Large head motion around the X-axis introduced a bulk B_0_ off-resonance difference at the back of the head and neck (Fig. 4C, D). This may have arisen due to the deformation of the subject’s soft tissue geometry after a large head movement, which significantly changes the shape of the body boundaries (skin folds and stretches) in the neck and jaw region. To improve the prediction precision for large motion around the X-axis, a subject-specific geometry mask depicting the anatomy in this new position was calculated at Position 4 from the magnitude image of measured field map. The remaining calculation steps in the simulation strategy remain the same. Owing to an adjustment of the subject’s geometry mask shape, both the simulation and the combined strategies were updated for large head movements.

### Multi-coil array B_0_ shimming simulation

To demonstrate the effectiveness of the proposed field map calculation methods, we compared the global shimming performance of measured field maps and the proposed calculated field maps.

We used a 16-channel multi-coil shimming array [28], the magnetic field from each shim coil was previously measured using a dual-echo GRE sequence (TE_1_/_2_ = 2.8/7.8 ms, TR = 15 ms, FOV = 208*208*160 mm^3^, FA = 12°) with 2 mm isotropic resolution. Shim currents were estimated by the MATLAB function ‘quadprog’ to minimize a cost function [29], which was defined as the sum of the residual magnetic field:

7 $$cost\_s= {\sum }_{voxel}{\left|{B}_{0}({\varvec{r}})-{B}_{0}^{shim}({\varvec{r}})\right|}^{2}$$

where ${B}_{0}^{shim}({\varvec{r}})$ denotes the magnetic field created by shim coils.

In our global shimming simulation, the cost function was evaluated over all voxels in the brain after down sampling their size from 1 to 2 mm isotropic, the maximal shim current on each coil was limited to 4A, and the maximal total shim current across all shim coils was limited to 50A. The calculated shim current for each shim coil was then applied to the measured shim coil basis map to generate a counteracting magnetic field to compensate for brain-induced B_0_ inhomogeneities.

During the simulation, we applied the calculated field maps to the cost function (Eq. 6), to replicate the situation where a measured field map is not available at a new head position. The generated shimming field, estimated from the calculated field map, was then used to shim the measured field map. For comparison, the optimal shimming currents for the measured field map were also calculated to generate a shimming field as the baseline. The shimming performance was evaluated by calculating the standard deviation of the measured field map before and after shimming (Vol. σB_0_).

## Results

**Fig. 5:**
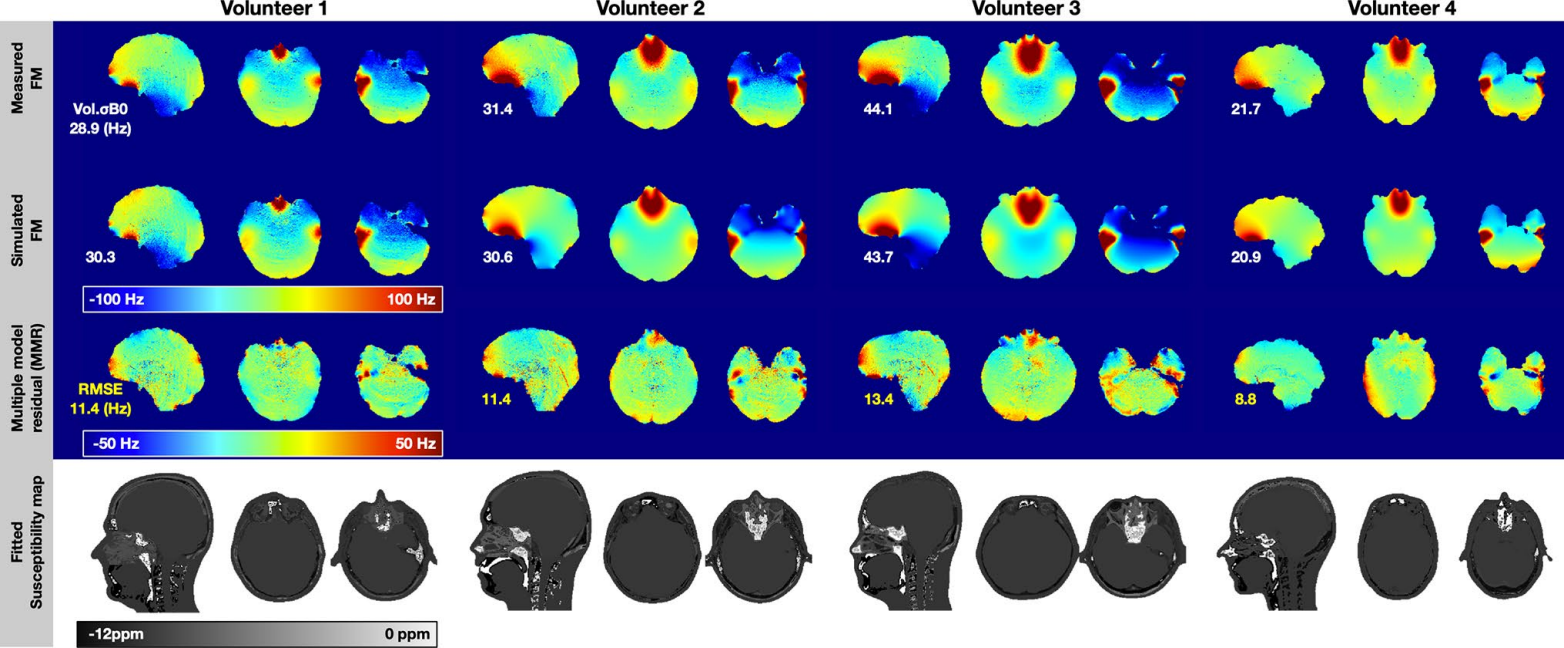
Four volunteers simulation results at the reference position. First row: Measured field maps, color range [− 100 Hz, 100 Hz]; Second row: Simulated field maps, color range [− 100 Hz, 100 Hz]; Third row: Residual field maps (Measured—Simulated), color range [− 50 Hz, 50 Hz]; Bottom row: Fitted susceptibility maps, color range [− 12 ppm, 0 ppm]

Figure 5 compares the simulation performance across four volunteers with the ground truth, measured FM. This demonstrates the success of field map prediction using the proposed method. Three representative slices are shown, with volume standard deviations (Vol.σB_0_) of the individual simulated FM and the measured FM listed on the left. The Vol.σB_0_ values for the simulated FM (ranging between 20.9 Hz to 43.7 Hz) are on average 3% different from the measured FM (ranging between 21.7 Hz and 44.1 Hz). The residual maps (Measured—Simulated) are shown with the RMSE value listed on the left. The average RMSE across the four volunteers is 11.2 Hz (ranging between 8.8 Hz to 13.4 Hz). The biggest discrepancies between the measured and simulated FMs were found around the (1) prefrontal cortex and temporal lobe regions, which are close to air-tissue interfaces, and (2) occipital lobe region, which is close to the superior sagittal sinus, straight sinus, and transverse sinus. The supplementary figure S3 compares the histogram and linear regression between the measured and simulated FMs. Quantitatively, an average linear regression slope of 0.94 (ranging between 0.91 to 0.97) was found, with Pearson correction coefficients R ≥ 0.93. The supplementary figure S4 summarizes the residual field map improvement from using the 3-class model susceptibility map to the proposed fitted susceptibility map, in short, a 30% average improvement was found (from 16.1 Hz to 11.2 Hz). The bottom row shows the fitted susceptibility map. The location of the sinuses and bony structures are in good agreement with the anatomical UTE image.

**Fig. 6:**
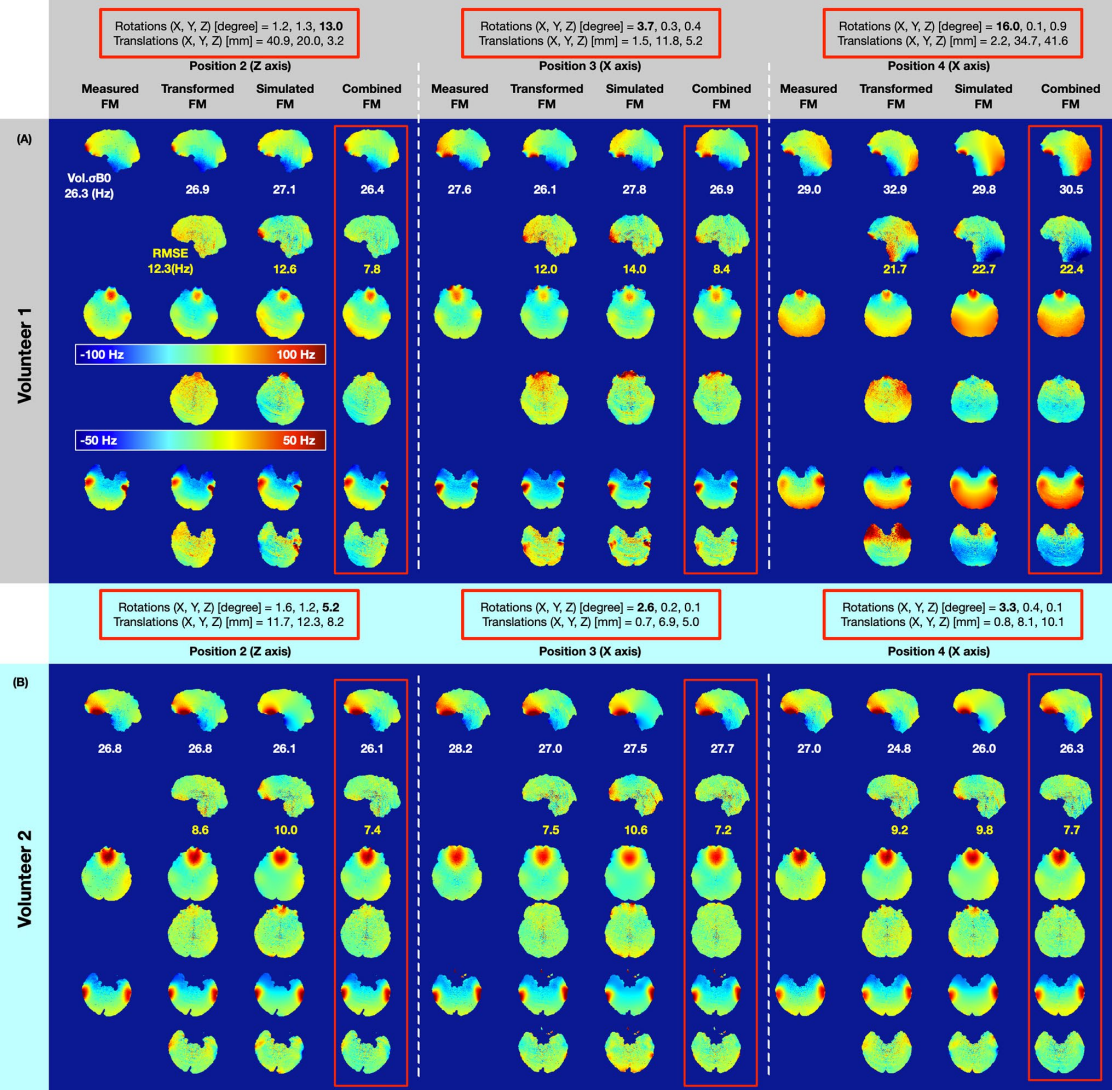
Motion induced field map estimation comparison in three representative slices from two subjects. The Vol. σB_0_ of the field map and RMSE of the estimated strategies were reported. The absolute rotation and translation values have been marked in red squares

Figure 6 compares the transformed FM, simulated FM, and combined FM (as shown in Fig. 4) with the measured FM in three representative axial slices from two volunteers, to show the prediction performance of these methods in motion induced B_0_ field changes. The combined FM yield improved prediction over the transformed FM for head rotations around the Z-axis and for a nodding movement around the X-axis. In Fig. 6A, volunteer 1 rotated the head about the Z-axis by 13 degrees. The RMSE is 12.2, 12.6, and 7.8 Hz for transformed FM, simulated FM, and combined FM, respectively. Compared with the transformed FM, the combined FM reduces the RMSE by 36% (from 12.2 Hz to 7.8 Hz). In Position 3, there is roughly a 4-degree head rotation around the X-axis (look down). The RMSE of the combined FM (8.4 Hz) was reduced by 30% with respect to the transformed FM (12.0 Hz). Because head rotation around the X-axis usually causes a realignment of the air-tissue interfaces with respect to B_0_. The significant B_0_ change happens in the frontal sinus, which is far away from the center of the head compared to the ethmoid and sphenoid sinus. With even larger head rotation around the X-axis, as shown in Position 4 (opposite direction, lookup), the RMSE for the transformed FM, simulated FM, and combined FM is 21.7, 22.7, and 22.4 Hz, respectively. All estimation strategies have larger RMSE values compared to Position 2 and Position 3, where a bulk B_0_ off-resonance difference was found at the posterior side. As shown in Fig. 6B, volunteer 2 had smaller head rotations in comparison to volunteer 1. Hence, the bulk B_0_ off-resonance difference in position 4 was not found. Comparison of measured FM, transformed FM, simulated FM, and combined FM for all four volunteers are shown in supplementary table ST1.

**Fig. 7:**
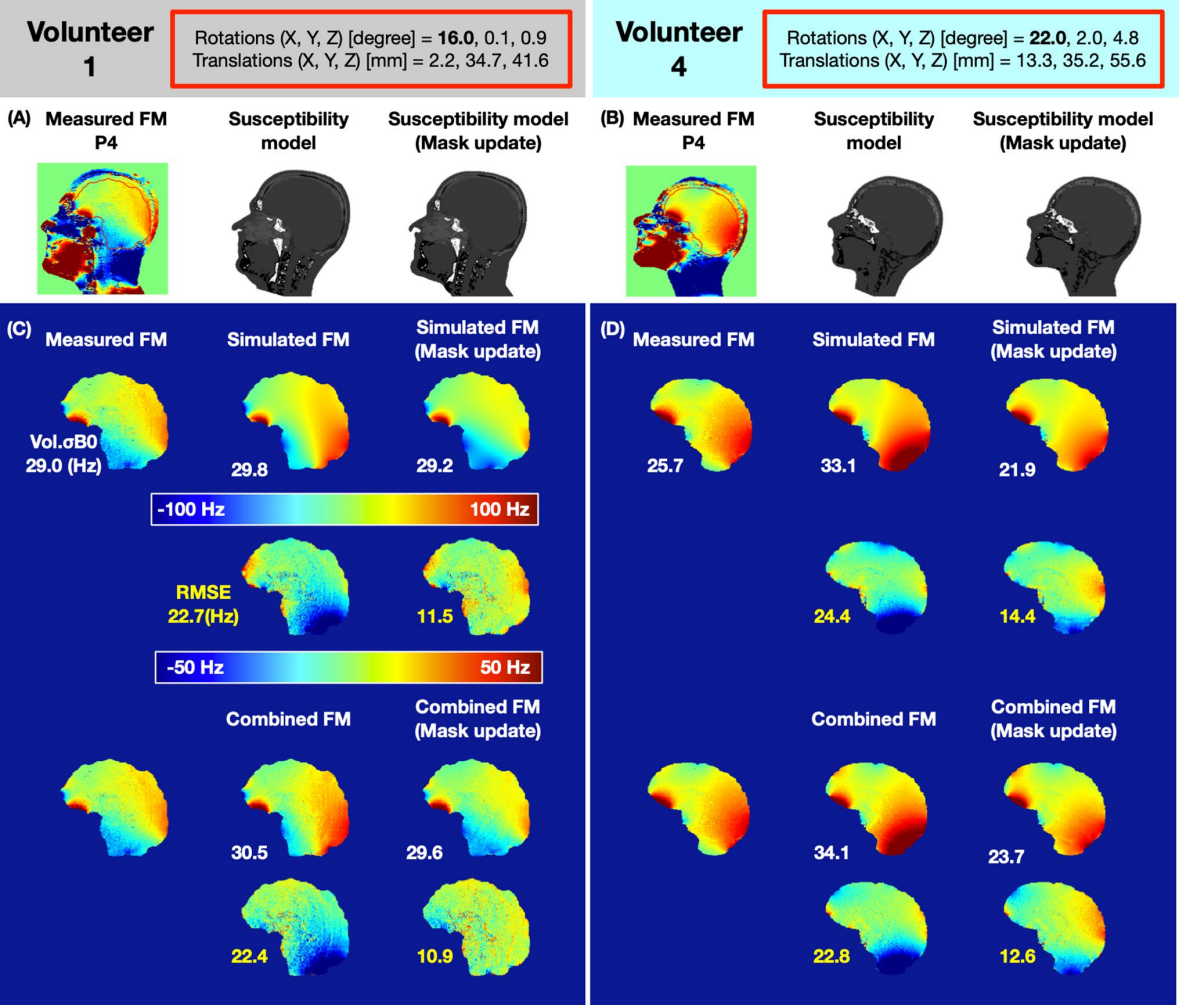
Mask updated susceptibility models used for Position 4 in two volunteers. **A** Mask of volunteer 1 tissue boundary update process. **B** Mask of volunteer 2 tissue boundary update process. **C** Compare the simulation improvement (RMSE) by introducing the new subject tissue boundary for volunteer 1 (the second row vs. the third row). **D** Compare the simulation improvement (RMSE) by introducing the new subject tissue boundary for volunteer 2 (the second row vs. the third row). The absolute rotation and translation values have been marked in red squares

In Fig. 6A, a large head motion around the X-axis introduced a bulk B_0_ off-resonance difference at the back of the head and neck. This may have arisen due to the deformation of the subject’s soft tissue geometry after a large head movement, which significantly changes the shape of the body boundaries (skin folds and stretches) in the neck and jaw region. Figure 7 shows the improvement of the simulated FM and combined FM after a large head movement owing to an adjustment of the subject’s geometry mask shape. The transformed FM, on the other hand, is not suitable for adapting to this adjustment. In Fig. 7A, B, a subject-specific geometry mask depicting the anatomy in this new position was calculated at Position 4 from the measured field map for volunteers 1 and 4, respectively. For volunteer 1, compared to the initial simulated FM without mask update in Fig. 7C (in the middle column), the simulated FM with mask update has reduced the RMSE by 50% (from 22.7 Hz to 11.5 Hz). The combined FM with mask update has further reduced the RMSE by 52% (from 22.9 Hz to 10.9 Hz) in comparison to the initial combined FM. For volunteer 4, compared to the initial simulated FM without mask update in Fig. 7D (in the middle column), the simulated FM with mask update has reduced the RMSE by 40% (from 24.4 Hz to 14.4 Hz). The combined FM with mask update has reduced the RMSE by 45% (from 22.8 Hz to 12.6 Hz) in comparison to the initial combined FM.

**Fig. 8:**
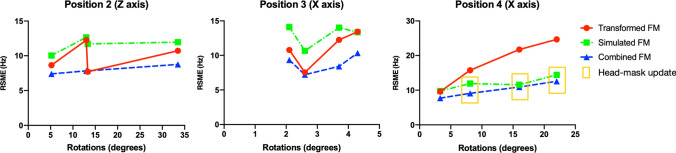
Comparison of prediction performance between the rigid-body transform, the simulation, and the combined strategies at multiple head angles. The yellow window indicates the simulation and combined strategies with subject head-mask update

Figure 8 shows how the prediction performance varies with the rotation angle for the rigid-body transform strategy, simulation strategy, and combined strategy at three head positions. For position 2, where rotations around the Z-axis were recorded up to 33 degrees, both the rigid-body transform strategy and the combined strategy have similar prediction performance, with an average of 10 Hz and 8 Hz RMSE from the measured FM, respectively. For position 3, averaging about 10 Hz and 8 Hz RMSE from the measured FM were also found for transform and combined strategies, respectively. However, the nodding-induced rotations around the X-axis (Position 3) are usually restricted by the head anatomical structure (approximately less than 5 degrees rotation). In comparison to the transform FM, the combined FM reduced the RMSE with respect to the ground-truth FM by 18.3%. For position 4, with opposite rotations around the X-axis (look up), a similar performance (about 10 Hz RMSE from the measured FM) was found for the transform and combined strategies, when the rotation angle is below an approximate magnitude of 5 degrees. We hypothesized that head motion with rotations above 5 degrees (approximately) will normally change the shape of the anatomy substantially, therefore, the prediction performance for the rigid-body transform strategy degrades. Nevertheless, after updating the subject head-mask, the simulation and combined strategies retain a similar prediction performance as at small rotations (approximately less than 5 degrees). By including the position-specific subject head-mask, the combined FM reduced the RMSE with respect to the ground-truth field map by 25.8% (Transform FM V.S. Combined FM).

**Fig. 9:**
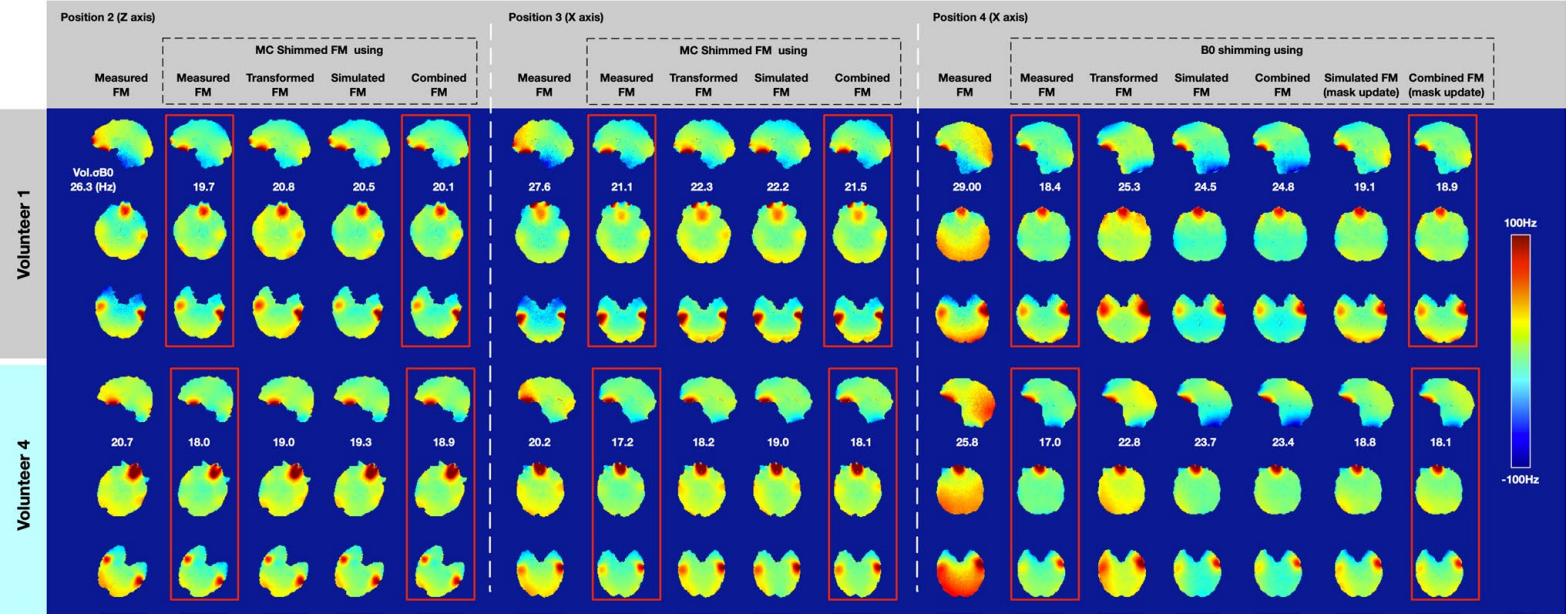
Global shimming simulation off-resonance magnetic field maps at three representative slices from two volunteers. For positions 2 and 3, the first column is the target measured FM for B_0_ shimming. The second column to the fifth column compared the residual field map after B_0_ shimming with proposed strategies, where the second column used the measured FM itself as the baseline. For position 4, additional B_0_ shimming results using the mask update susceptibility model is compared with the B_0_ shimming result using measured field FM

Figure 9 exemplifies how the proposed strategy can be used for future “real-time” shimming. In each head position, the first column shows the measured field map, which is considered to be the B_0_ shimming target. The shim coil setup, as well as simulation parameters, are shown in supplementary figure S5A. For the B_0_ shimming experiment, the shim performance results are shown in the second to fifth columns. The baseline condition is to use the measured field map as shimming targeted. For position 2 of volunteer 1, global B_0_ shimming with a measured FM could reduce Vol.σB_0_ by 25% (from 26.3 Hz to 19.7 Hz). The shim performance using the rigid-body transformed FM, simulation FM, and combined FM as a B_0_ shimming condition, reduced the Vol.σB_0_ of the measured FM by 21% (to 20.8 Hz), 22% (to 20.5 Hz), and 24% (to 20.1 Hz), respectively. The shim performance using the proposed field map calculation strategies are similar, with the combined strategy slightly closer to the baseline. The spatial distributions of the residual field map after global shimming using the combined FM are comparable to the one shimmed with the measured FM in the new position. In position 4, the head rotation is relatively large for both volunteers 1 and 4, and the reduction of Vol.σB_0_ for the proposed rigid body transform FM, simulation FM, and combined FM is 13% (from 29.0 Hz to 25.3 Hz), 15% (from 29.0 Hz to 24.5 Hz), and 14% (from 29.0 Hz to 24.8 Hz) respectively, whereas the baseline is 36% (down to 18.4 Hz). The bulk B_0_ off-resonance difference at the back of the head and neck (as shown in Fig. 6 and Fig. 7) leads to an incorrect B_0_ shimming calculation. With the adjustment of the subject’s geometry mask shape, in the case of volunteer 1, the Vol.σB_0_ of the residual field map using the simulated FM and combined FM with mask reduced the inhomogeneity by 22% (from 24.5 Hz to 19.1 Hz) and 23% (24.8 Hz to 18.9 Hz), respectively. For volunteer 4, the Vol.σB_0_ of the residual field map using the simulated FM and combined FM with mask update was reduced by 2% (from 23.7 Hz to 18.8 Hz) and 23% (23.1 Hz to 18.1 Hz), respectively. Supplementary figure S5B provides simulation results for all four subjects.

## Discussion

In this work, we proposed a ΔB_0_ field simulation strategy using a fitted subject-specific multi-class tissue susceptibility model generated from a UTE image and a reference field map. We verified the prediction error of our approach to validate it for the case that an experimentally measured field map is not available. Compared with the rigid-body transformed field map, the proposed simulation strategy (combined FM) based on local susceptibility effects and position-specific masking achieved superior prediction performance at multiple head positions.

Substantial discrepancies in these methods were found when predicting motion induced B_0_ changes with a large head motion around the X-axis. However, with additional information from subject anatomy in the new head position, the simulation strategy and combined strategy were able to mitigate this discrepancy.

For the proposed simulation strategy, there are a few considerations that determine the accuracy of the simulated field map:

### The Fourier approximation method

We adopted the Fourier based dipole-approximations method [13, 14], which enables rapid computation of the magnetic field, using a high sampling resolution and spatial padding factor. The spatial padding extends the computational volume outside the imaged FOV, to make sure that the perturbing induction fields have diminished at the computational volume boundaries, avoiding artifacts like folding over of the field into the opposite computational volume side. This is primarily due to the constraints on the k-space scaling factor in Eq. 2 which is singular at ***k*** = 0. along with the periodic nature of the Discrete Fourier Transformation (DFT). It is also important to stress that low-resolution sampling limits computational accuracy through misrepresentations of the compartment borders as well as the k-space scaling coefficient (i.e., insufficient sampling of the dipole response in k-space due to low grid resolution) in Eq. 2 [15, 30].

Based on previous research [17, 30], the pitfalls of the Fourier dipole approximation method could be carefully mitigated through high spatial sampling (with voxel size being less than 1 mm isotropic) and the spatial padding factor (greater than 3).

### The measured field map

We used the measured field maps as a ground truth B_0_ map for the proposed simulation. However, it is difficult to acquire an unbiased B_0_ map in the brain, particularly in voxels near the air-tissue interface where signal loss from intra-voxel dephasing and extreme phase wrapping behavior make accurate B_0_ measurements very challenging. In addition to that, the phase offset caused by eddy currents is another source of disagreement between the measured and simulated field maps. Instead of the vendor-provided dual-echo 2D GRE, we used a dual-echo 3D GRE sequence for field mapping. We tried to minimize the readout and slice-selective gradient eddy currents by using monopolar readout and non-selective excitation. Unfortunately, several factors cannot be modeled properly, including (1) Noise in the phase image and (2) the “background” field that compensates for field contributions due to the receive coil and cushion mats, etc. We introduced the residual FM (MMR) at the reference position to account for such factors. We found that other field sources, specifically the effect from eddy currents in the phase encoding and readout directions of the field map sequence, only play marginal effects, as estimated with a phantom scan (supplementary figure S7-8). It can be noted that such phantom-based eddy current estimation can be inaccurate, in case large motion causes the head to be located outside the pre-defined phantom region. Therefore, we decided not to include any corrections for eddy-current induced effects.

In the present study, we observed a linear field map contribution (from anterior to posterior, the phase-encoding direction) within the measured field map, that was present after demodulating the simulated SH shim field and sample-induced Bχ field (Fig. 2C). Without correction of that effect, the dipole fitted to take into account sources outside the body, would shift towards the posterior part of the head and then fall outside the body region—a physiologically not meaningful result. Instead, if we used k-space shifts modeling to estimate the residual field to its first order, an idea previously explored by Diefenbach et al. [31] the dipole was correctly located within body in agreement with previous studies (Dienfenbach et al. [31], Koch et al. [17]). As shown in supplementary figure S9-S11, this approach diminished the discrepancies between the measured and simulated field map. There may be many reasons behind this phase-error term. We hypothesize that the main factor is a field deviation originating from imperfections in the vendor-calibrated “Tune-up shim”, as shown in supplementary figure S10 and supplementary table ST2. For future work we suggest to pairing the actual tune-up shim coefficients with the measurement to scale the SH shim fields.

### Tissue susceptibility models

The accuracy of the subject-specific susceptibility model for discriminating air-bone boundaries plays a critical role for the quality of our field map simulations. The temporal and the nasal region are composed of complex structures comprising soft tissue, mucus, air, and cartilage, which usually result in strong partial-volume effects. It is challenging to fully resolve the different tissue types with the spatial resolution used in this work. To circumvent tissue misclassification, we propose an iterative, nonlinear optimization algorithm to determine susceptibility values for each segment of the tissue map obtained from UTE. The optimization algorithm aims to diminish the residual error (SMR) within the brain mask (Fig. 2A–D). The proposed iterative nonlinear optimization algorithm is similar to a previously published phase replacement method [19], which was used to determine the susceptibility values of air, bone, and teeth. Both algorithms require a segmented air and bone map and phase (frequency) information around the structure of interest. Compared with the phase replacement method, where the segmented air and bone masks were obtained from a short echo GRE image, our multiple segment model is based on the inverse log-scaled UTE image, which facilitates identification of air-tissue and air-bone boundaries.

In contrast to a previously published method [17] which requires anatomical data with CT and MRI, the use of a UTE sequence provides adequate anatomical information for segmentation of air/bone boundaries in the human head. As a result, the need for CT images to detect bone boundaries can be addressed with an MR scanner. The UTE sequence gives an analogous histogram distribution to the inverse log-scaled ZTE images, introduced by Wiesinger et. al [21]. We use a flip-angle of 1 degree to suppress signals from both water and fat to provide a uniform soft-tissue contrast (PD-like) and therefore detection of the bone. The low-flip angle (smaller than the Ernst angle) also avoids T1 saturation of long T1 components (e.g., CSF and eyes) that can lead to misclassification as bony structures. Compared with previously proposed UTE methods [32, 33] using T2 relaxation differences, the short TE and low flip-angle UTE take advantage of PD contrast and do not require long T2 or T1 suppression, like echo subtraction or inversion pulses.

The actual susceptibility can differ between head regions, although the tissues still have similar -log (UTE) intensities. For instance, the mastoid cavity was reported to have lower susceptibilities than the rest of the sinuses [19]. In principle, the susceptibility value could be determined for each individual voxel within the multiple segment model. However, estimating the susceptibility value voxel-by-voxel is time consuming and may be impractical. To obtain a high-quality field map estimation, one can decrease the bin-size to have a finer segment model or allow different susceptibility values for tissues with similar -log (UTE) intensities. We compared both approaches in supplementary figure S6B, where we found that the use of VOI and VOI-specific estimates of the susceptibility for each tissue class improved estimation. Without spatially defined VOIs, the multi-class tissue model only reduced the field map error by 3% (from 15.9 Hz to 15.6 Hz), whereas a multi-class tissue model with 4 spatially defined VOIs could reduce the residual error by 20% (from 15.9 Hz to 12.6 Hz) and 8 VOIs by 28% (from 15.9 Hz to 11.4 Hz).

The proposed iterative nonlinear optimization algorithm is able to approximate the susceptibilities for various tissue classes, however, the approximation accuracy at this stage is still rudimentary in comparison to the previously published phase replacement method [19]. Further investigations, including a comprehensive frequency map for the fitted susceptibility model (field map of the whole head, instead of the brain), are needed to further improve approximation accuracy for future applications.

### Motion induced field map variations

As shown in Fig. 6 and Fig. 7, estimating a field map at a new head position using the rigid-body transform strategy and corresponding motion parameters (6 DOFs) from image-based retrospective motion correction provides a simple and good approximation for small motion induced ΔB_0_ variants (approximately < 5 degrees). Nonetheless, the rigid-body transform strategy is insufficient to predict the brain field map due to large motion. The field map error becomes particularly large in the jaw and neck regions. Our extended model showed that ΔB_0_ shifts arise from the displacement of the jaw and neck, leading to alterations of the ΔB_0_ field in the brain, especially in its inferior regions. Compared to the rigid-body transform strategy, the combined field map strategy has a similar performance in predicting the field map for small movements.

### Future implementation

The current simulation work builds the foundation for “real-time” B_0_ shimming to be carried out in the future. It is intended for combination with a multi-coil B_0_ shimming array [28] and a motion camera system [34] to achieve “real-time” B_0_ shimming. However, a description and evaluation of the additional hardware and software integration necessary to achieve this goal is beyond the scope of the current work. For future implementation of a suitable head model, we feel that a mixed-matter deformation model for the head, including information about its elastic properties, could allow more precise predictions about the field map at different head positions without requiring additional measurements. This suggestion is highly interesting but not trivial to solve either in terms of the mathematical model, its parameters and online calculation. Possibly it can suffice to use low resolution rapid MRI to capture the shape and position of the head to obtain sufficient information to achieve a calculation of the field distribution. Such an approach could be more clinically/real-time applicable since it would require only a brief MRI acquisition yet simplify the modeling aspect.

In conclusion, we found that a rigid-body transformation of a measured field map provides a feasible approximation for motion induced ΔB_0_ variants. The proposed field map simulation strategy can estimate the motion induced ΔB_0_ changes without MR measurement. A further improvement was observed by taking the actual position and subject geometry into account. This modeling strategy may be used for improved image reconstruction, better (dynamic) B_0_ shimming and B_0_ related artifacts correction in cases where no measured field map is available for the different head positions.

## Supplementary Information

Below is the link to the electronic supplementary material.

Supplementary file1 (DOCX 10048 KB)

## Data Availability

The data that support the findings of this study are available on the request of corresponding auther JZ.

## References

[1] Jezzard P, Clare S (1999). Sources of distortion in functional MRI data. Hum Brain Mapp.

[2] Glover GH (1999). 3D Z-shim method for reduction of susceptibility effects in BOLD fMRI. Magn Reson Med.

[3] Stenger VA, Boada FE, Noll DC (2000). Three-dimensional tailored RF pulses for the reduction of susceptibility artifacts in T2/(*)-weighted functional MRI. Magn Reson Med.

[4] Jezzard P, Balaban RS (1995). Correction for geometric distortion in echo planar images from b0 field variations. Magn Reson Med.

[5] Stockmann JP, Wald LL (2018). In vivo B0 field shimming methods for MRI at 7 T. Neuroimage.

[6] Zaitsev M, Akin B, LeVan P, Knowles BR (2017). Prospective motion correction in functional MRI. Neuroimage.

[7] Lamberton F, Delcroix N, Grenier D, Mazoyer B, Joliot M (2007). A new EPI-based dynamic field mapping method: Application to retrospective geometrical distortion corrections. J Magn Reson Imaging.

[8] Dymerska B, Poser BA, Barth M, Trattnig S, Robinson SD (2018). A method for the dynamic correction of B0-related distortions in single-echo EPI at 7 T. Neuroimage.

[9] Dymerska B, Poser BA, Bogner W, Visser E, Eckstein K, Cardoso P, Barth M, Trattnig S, Robinson SD (2016). Correcting dynamic distortions in 7T echo planar imaging using a jittered echo time sequence. Magn Reson Med.

[10] Alhamud A, Taylor PA, van der Kouwe AJW, Meintjes EM (2016). Real-time measurement and correction of both B0 changes and subject motion in diffusion tensor imaging using a double volumetric navigated (DvNav) sequence. Neuroimage.

[11] Wallace TE, Polimeni JR, Stockmann JP, Hoge WS, Kober T, Warfield SK, Afacan O (2021). Dynamic distortion correction for functional MRI using FID navigators. Magn Reson Med.

[12] Durrant CJ, Hertzberg MP, Kuchel PW (2003). Magnetic susceptibility: Further insights into macroscopic and microscopic fields and the sphere of Lorentz. Concepts Magn Reson Part A Bridg Educ Res.

[13] Salomir R, de Senneville BD, Moonen CTW (2003). A fast calculation method for magnetic field inhomogeneity due to an arbitrary distribution of bulk susceptibility. Concepts Magn Reson.

[14] Marques JP, Bowtell R (2005). Application of a Fourier-based method for rapid calculation of field inhomogeneity due to spatial variation of magnetic susceptibility. Concepts Magn Reson Part B Magn Reson Eng.

[15] Boegle R, MacLaren J, Zaitsev M (2010). Combining prospective motion correction and distortion correction for EPI: Towards a comprehensive correction of motion and susceptibility-induced artifacts. Magn Reson Med Phy, Biol Med.

[16] Jenkinson M, Wilson JL, Jezzard P (2004). Perturbation method for magnetic field calculations of nonconductive objects. Magn Reson Med.

[17] Koch KM, Papademetris X, Rothman DL, De Graaf RA (2006). Rapid calculations of susceptibility-induced magnetostatic field perturbations for in vivo magnetic resonance. Phys Med Biol.

[18] Neelavalli J, Cheng YCN, Jiang J, Haacke EM (2009). Removing background phase variations in susceptibility-weighted imaging using a fast, forward-field calculation. J Magn Reson Imaging.

[19] Buch S, Liu S, Ye Y, Cheng YC, Neelavalli J, Haacke EM (2015). Susceptibility mapping of air, bone, and calcium in the head. Magn Reson Med.

[20] Shi Y, Vannesjo SJ, Miller KL, Clare S (2018). Template-based field map prediction for rapid whole brain B0 shimming. Magn Reson Med.

[21] Wiesinger F, Sacolick LI, Menini A, Kaushik SS, Ahn S, Veit-Haibach P, Delso G, Shanbhag DD (2016). Zero TE MR bone imaging in the head. Magn Reson Med.

[22] Tustison NJ, Avants BB, Cook PA, Zheng Y, Egan A, Yushkevich PA, Gee JC (2010). N4ITK: improved N3 bias correction. IEEE Trans Med Imaging.

[23] Smith SM (2002). Fast robust automated brain extraction. Hum Brain Mapp.

[24] Smith SM, Jenkinson M, Woolrich MW, Beckmann CF, Behrens TE, Johansen-Berg H, Bannister PR, De Luca M, Drobnjak I, Flitney DE, Niazy RK, Saunders J, Vickers J, Zhang Y, De Stefano N, Brady JM, Matthews PM (2004). Advances in functional and structural MR image analysis and implementation as FSL. Neuroimage.

[25] Deistung A, Schafer A, Schweser F, Biedermann U, Turner R, Reichenbach JR (2013). Toward in vivo histology: a comparison of quantitative susceptibility mapping (QSM) with magnitude-, phase-, and R2*-imaging at ultra-high magnetic field strength. Neuroimage.

[26] Wharton S, Bowtell R (2010). Dipole-based filtering for improved removal of background field effects from 3D phase data. Proc Intl Soc Magn Reson Med..

[27] Jenkinson M, Bannister P, Brady M, Smith S (2002). Improved optimization for the robust and accurate linear registration and motion correction of brain images. Neuroimage.

[28] Aghaeifar A, Mirkes C, Bause J, Steffen T, Avdievitch N, Henning A, Scheffler K (2018). Dynamic B0 shimming of the human brain at 9.4 T with a 16-channel multi-coil shim setup. Magn Reson Med.

[29] Zhou J, Stockmann JP, Arango N, Witzel T, Scheffler K, Wald LL, Lin FH (2020). An orthogonal shim coil for 3T brain imaging. Magn Reson Med.

[30] Cheng YCN, Neelavalli J, Haacke EM (2009). Limitations of calculating field distributions and magnetic susceptibilities in MRI using a Fourier based method. Phys Med Biol.

[31] Diefenbach MN, Ruschke S, Eggers H, Meineke J, Rummeny EJ, Karampinos DC (2018). Improving chemical shift encoding-based water-fat separation based on a detailed consideration of magnetic field contributions. Magn Reson Med.

[32] Keereman V, Fierens Y, Broux T, De Deene Y, Lonneux M, Vandenberghe S (2010). MRI-based attenuation correction for PET/MRI using ultrashort echo time sequences. J Nucl Med.

[33] Catana C, van der Kouwe A, Benner T, Michel CJ, Hamm M, Fenchel M, Fischl B, Rosen B, Schmand M, Sorensen AG (2010). Toward Implementing an MRI-Based PET Attenuation-Correction Method for Neurologic Studies on the MR-PET Brain Prototype. J Nucl Med.

[34] Zaitsev M, Dold C, Sakas G, Hennig J, Speck O (2006). Magnetic resonance imaging of freely moving objects: prospective real-time motion correction using an external optical motion tracking system. Neuroimage.

