# Supplementary material for: Prediction of motion induced magnetic fields for human brain MRI at 3 T
Source: MAGMA. 2023 Mar 25;36(5):797–813. doi: 10.1007/s10334-023-01076-0 (PMC10504152; doi:10.1007/s10334-023-01076-0)
Supplement: Supplementary file 1 — Supplementary file1 (DOCX 10048 KB) [file 10334_2023_1076_MOESM1_ESM.docx]

**Supplementary document:**


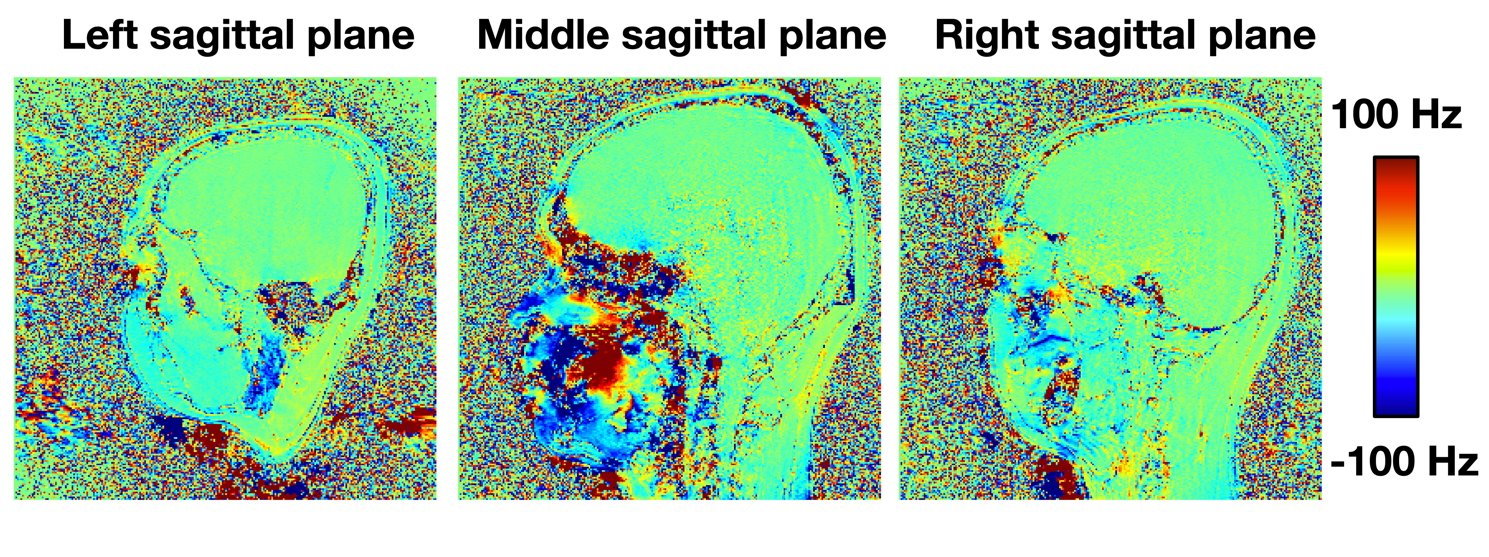
 **Fig. S1** Sagittal slices for calculated fat field map. The chemical shift field map was calculated through a two-point Dixon like field map strategy, where two field maps were acquired. The first field map was acquired at both echo times set with the water and fat resonances to be in-phase (in.-TE1/2 = 2.68/7.49 ms), and the latter both at opposite-phase echo time (opp.-TE1/2 = 3.70/8.61 ms). Subtracting opposite-phase from in-phase field map removes most ΔB_0_ effect from sample susceptibility induced and SH shim, leaving the chemical shift frequency difference caused by the fat tissue.


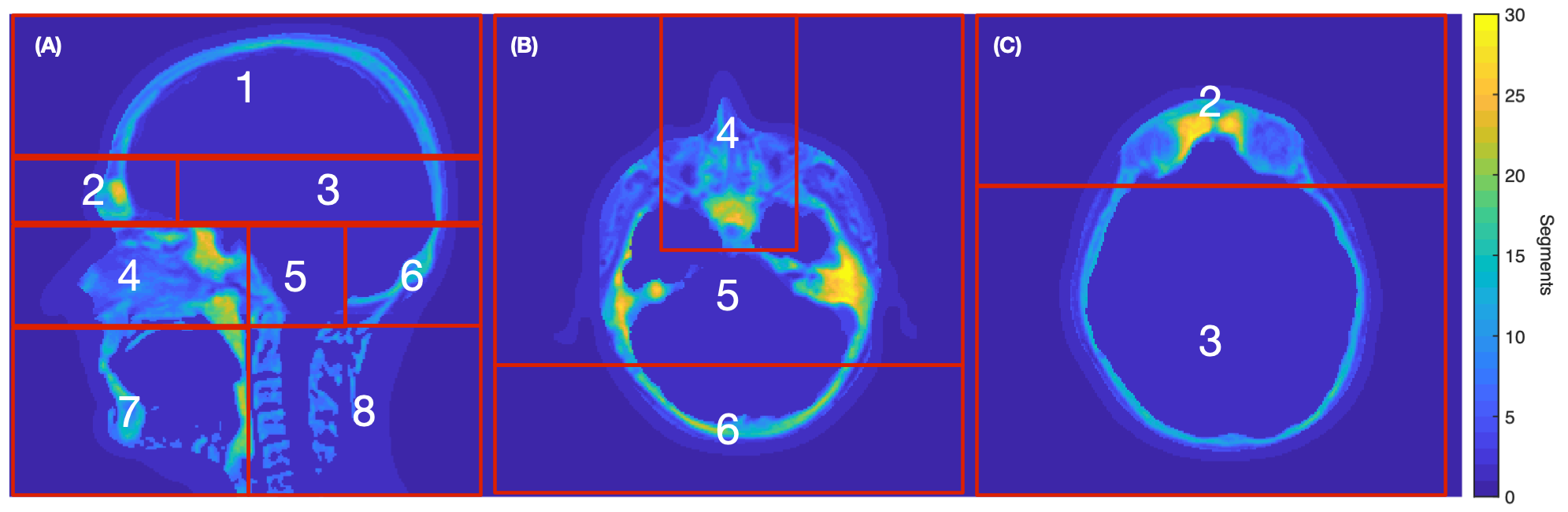


**Fig. S2** Three representative slices of multiple segment model with eight VOIs for volunteer 1.


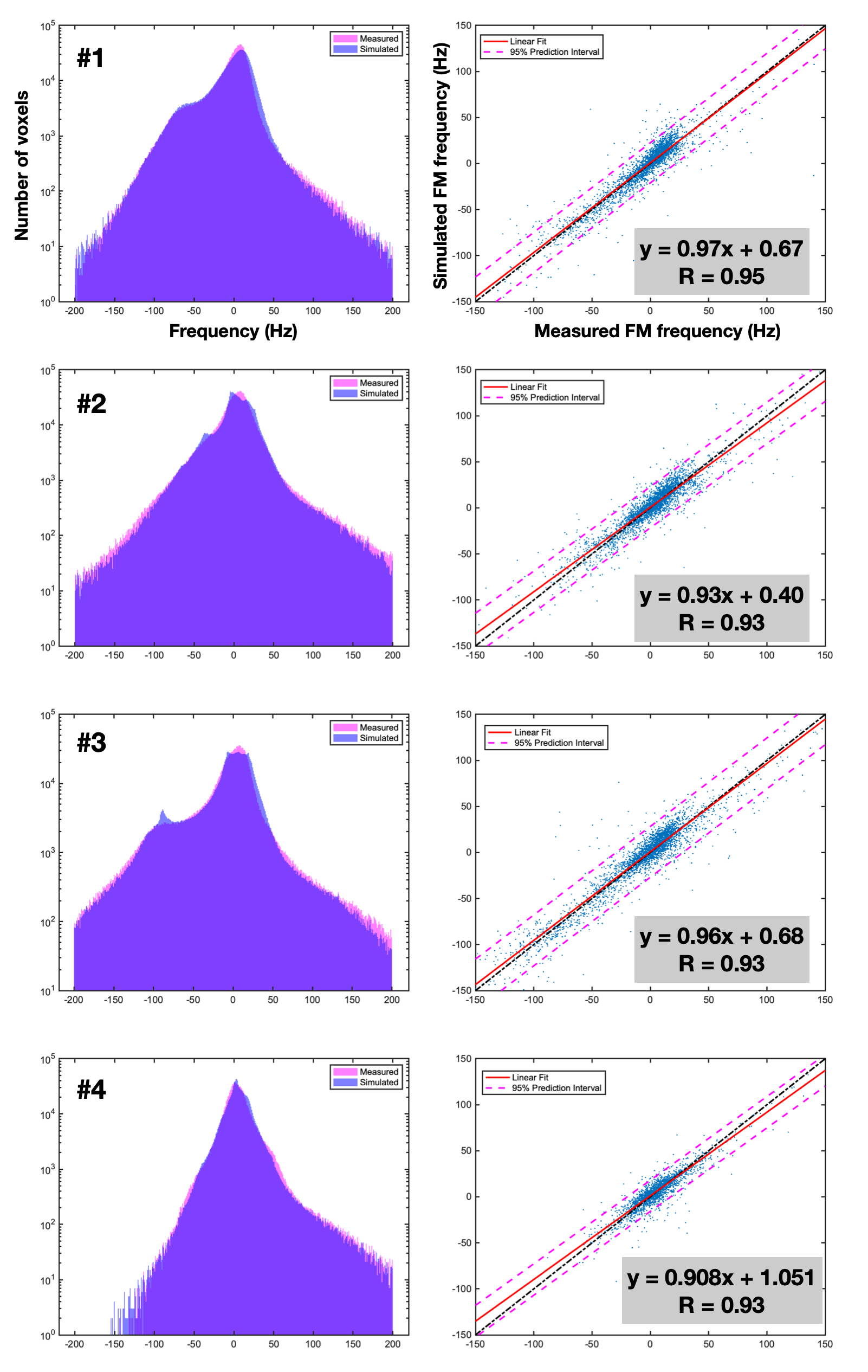


**Fig. S3** Comparison between measured and simulated ΔB_0_ maps in four volunteers. The measured and simulated ΔB_0_ field maps are confronted one another through superimposed histograms (Left) and linear regressions (Right)


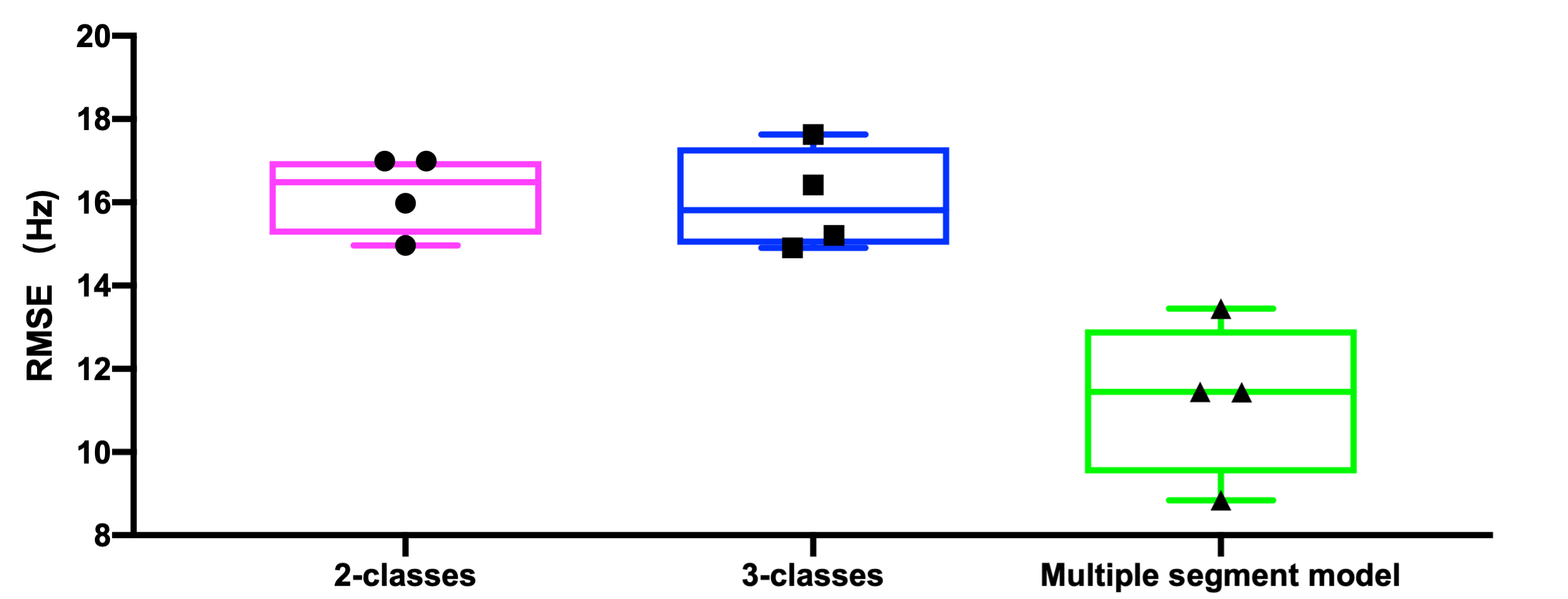


**Fig. S4** Comparison of the RMSE values for three head models over four volunteers. The mean, minimal and maximal values were shown.


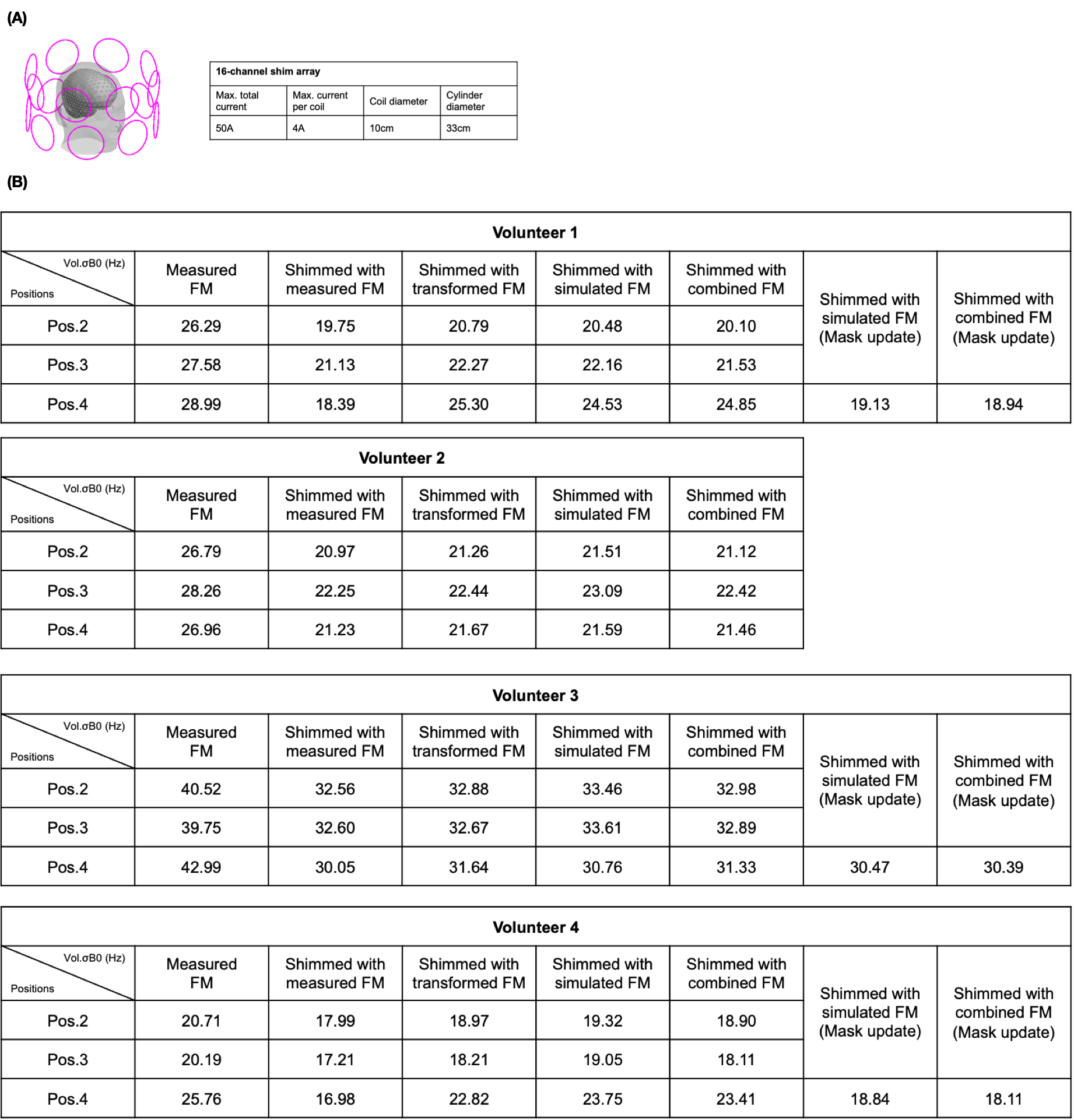


**Fig. S5** Multi-coil B_0_ shimming simulations for all four volunteers. (A) Multi-coil setup and simulation current limits. (B) B_0_ shimming results.


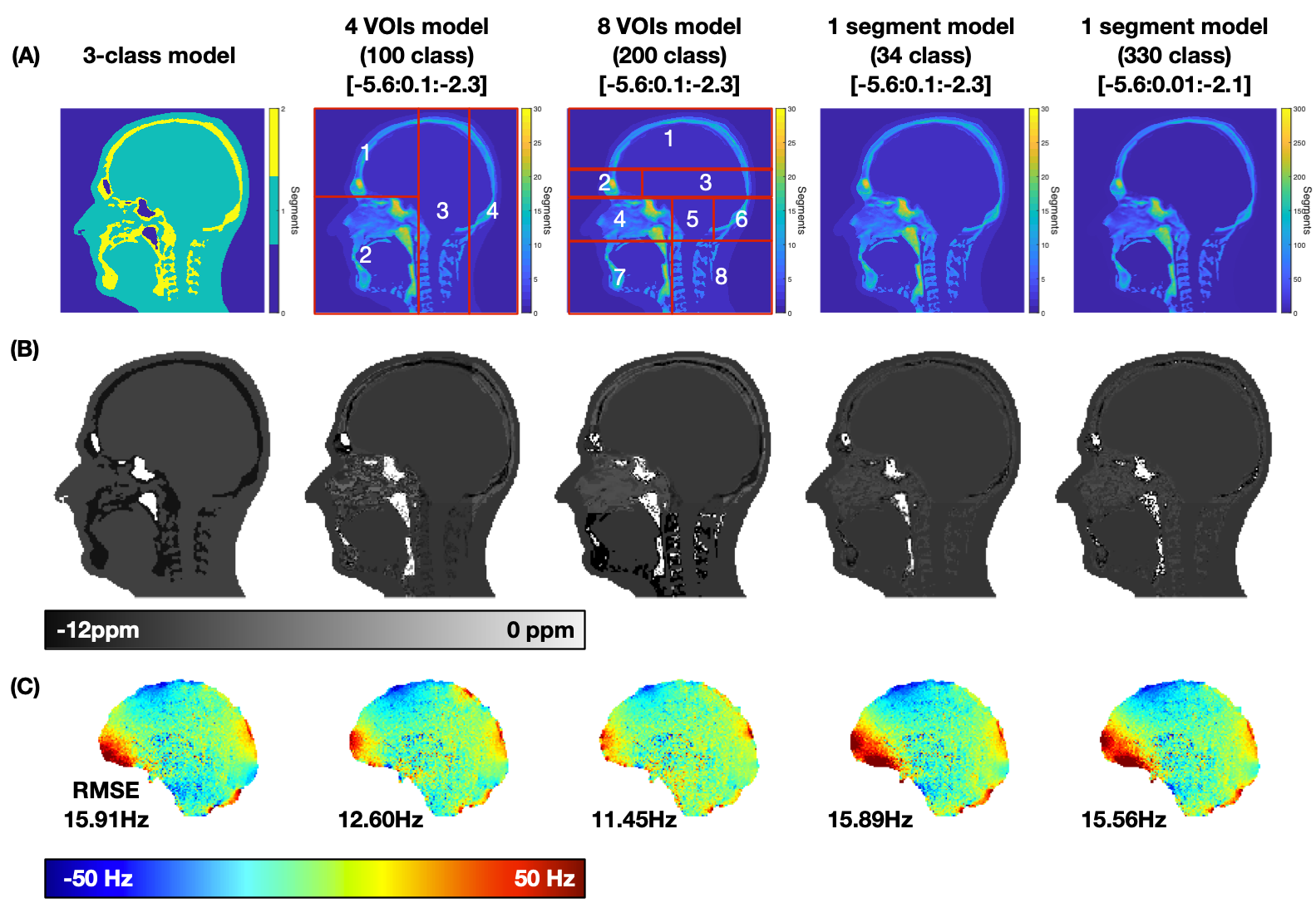


**Fig. S6** Comparison of the RMSE values for five segment models, (A) from left to right are 3-classes model, 4VOIs model (where 4 VOIs have been defined for a 1segement model with 34 tissue classes), 8VOIs model (where 8 VOIs have been defined for a 1segement model with 34 tissue classes), 1segement model with 34 tissue classes, and 1segement model with 330 tissue classes. (B) RMSE comparison, where the 3-classes model is the baseline. (C) Corresponding susceptibility maps.


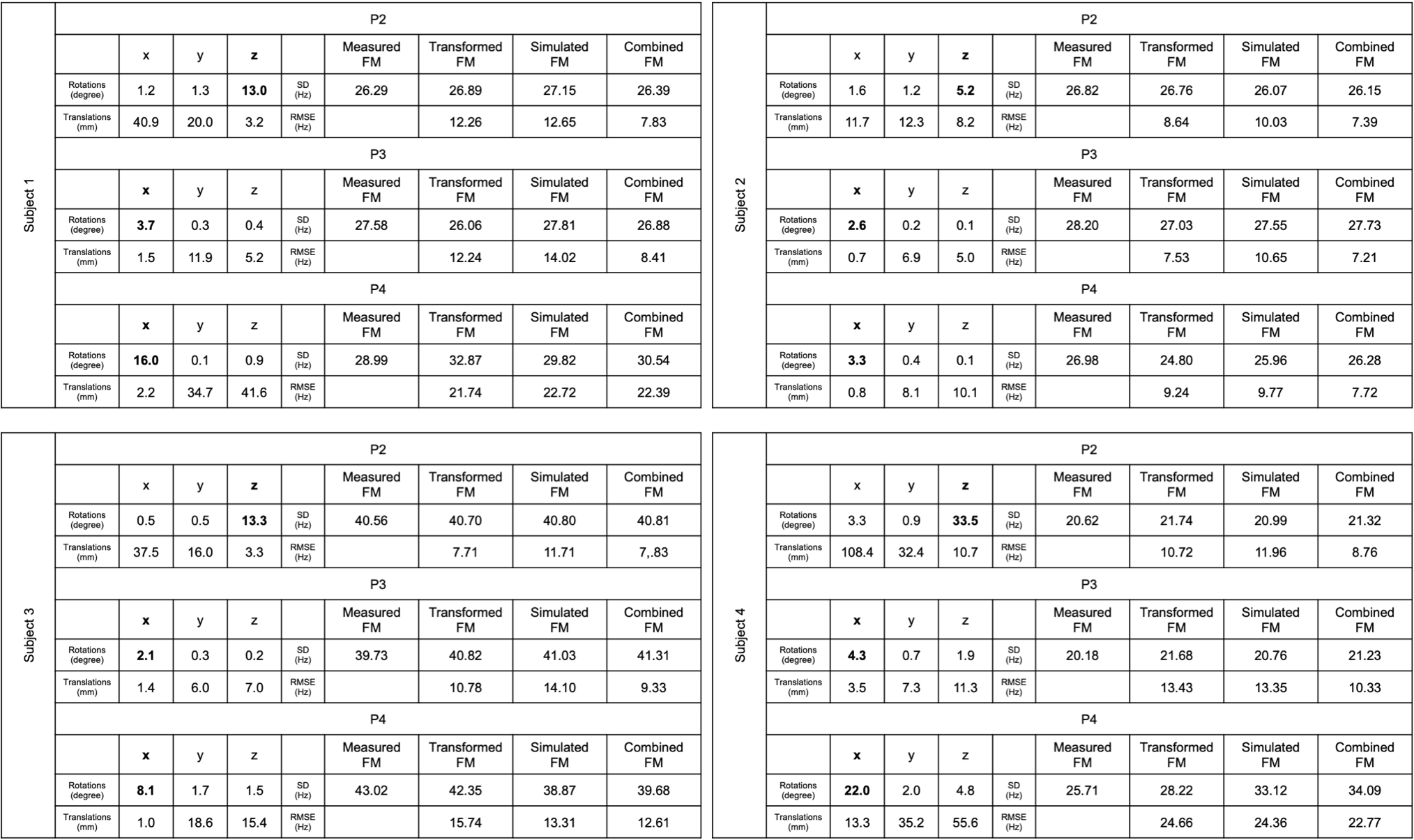


**ST.1** Comparison of measured FM, transformed FM, simulated FM, and combined FM data for 4 volunteers.


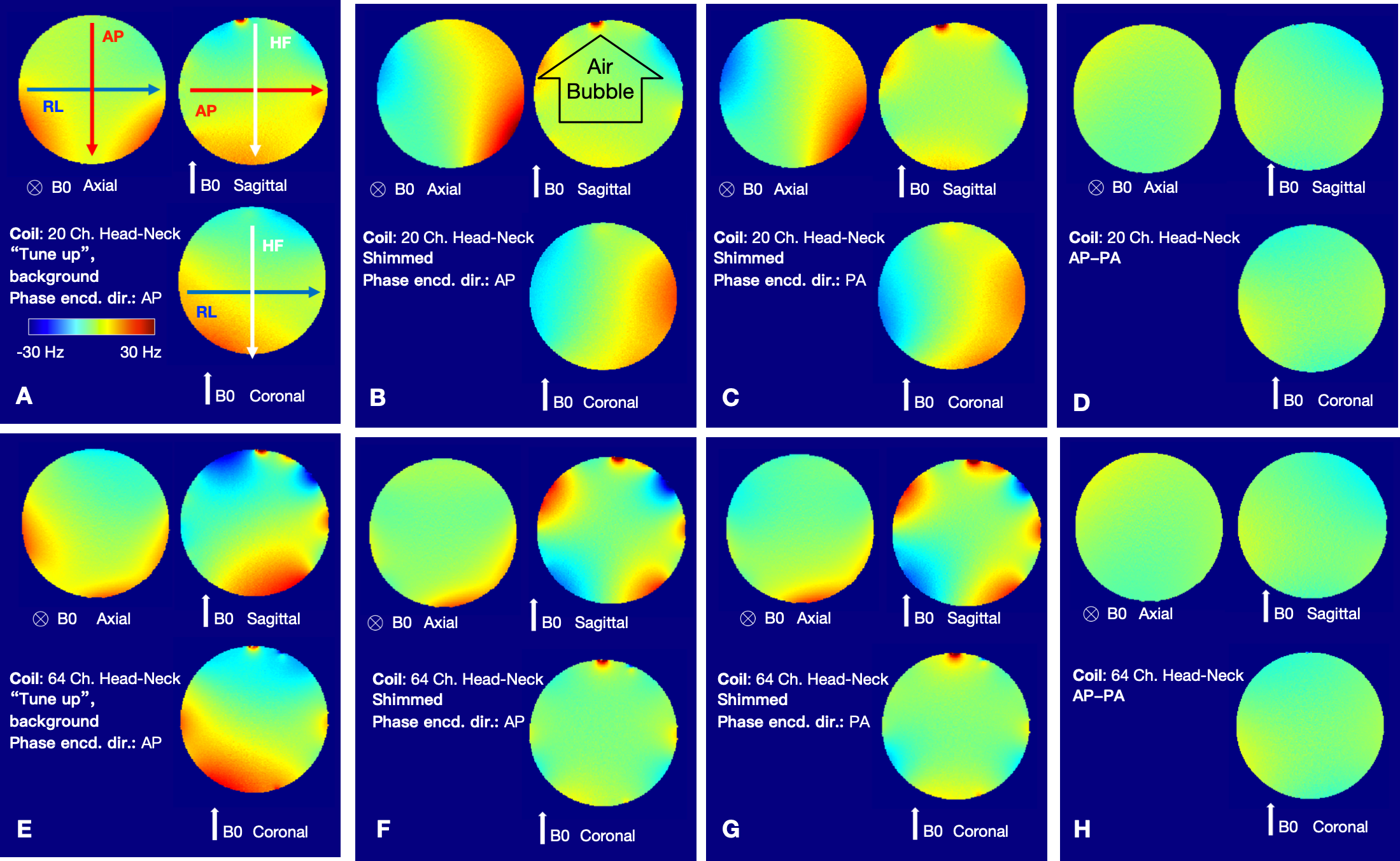


**Fig. S7** Three orthonormal views of 3D GRE field maps obtained on a spherical phantom (A) Measured background without target-specific shim (“Tune-up shim” only). (B) Measured field map with phase encoding direction from the Anterior to the Posterior, target-specific shim has been applied. (C) phase encoding direction from the Posterior to Anterior, the shim parameters are the same as in (B). (D) The difference of the measured field maps with opposite phase encoding directions, indicating the contribution from eddy currents of the Y gradient. The A, B, and C are measured using the 20-channel head-neck receive coil. The E, F, and G are corresponding field maps with the same protocol to A, B, C, respectively, using 64-channel head-neck receive coil.


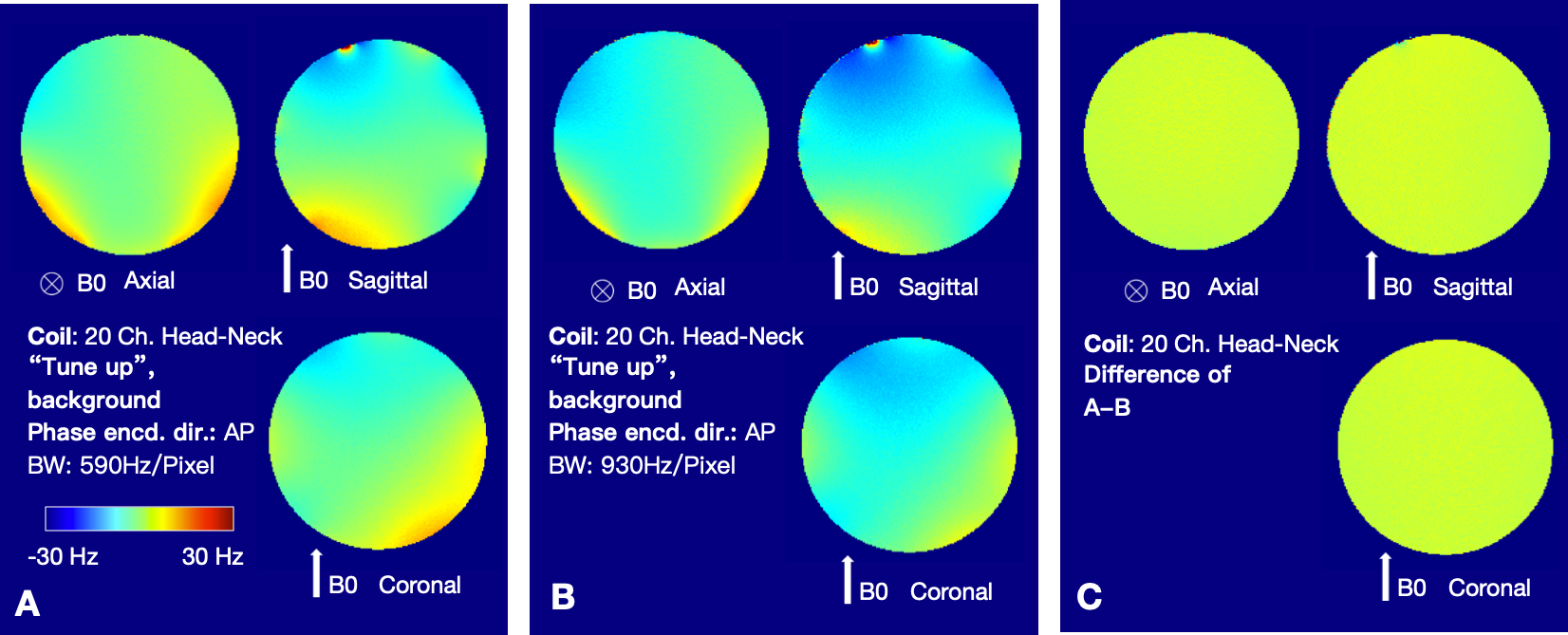


**Fig. S8** Three orthonormal views of 3D GRE field maps obtained on a spherical phantom (A) Read-out bandwidth set to be 590Hz/Pixel. (B) Read-out bandwidth set to be 930Hz/Pixel. (C) The difference of the measured field maps A-B.

**Potential sources of the observed field shifts in the AP direction:**

In the measured field map as shown in Figure 2, we observed an additional field contribution which shifts the location of the fitted dipole source from Z-axis direction to a diagonal direction. This effect can result in dipole source outside the body region - a physiologically not meaningful location. We hypothesize this might be due to a field deviation originating from the vendor-calibrated shim determined in a spherical phantom (known as the “Tune-up shim”) We explored this hypothesis through numerical simulations and subject measurements.


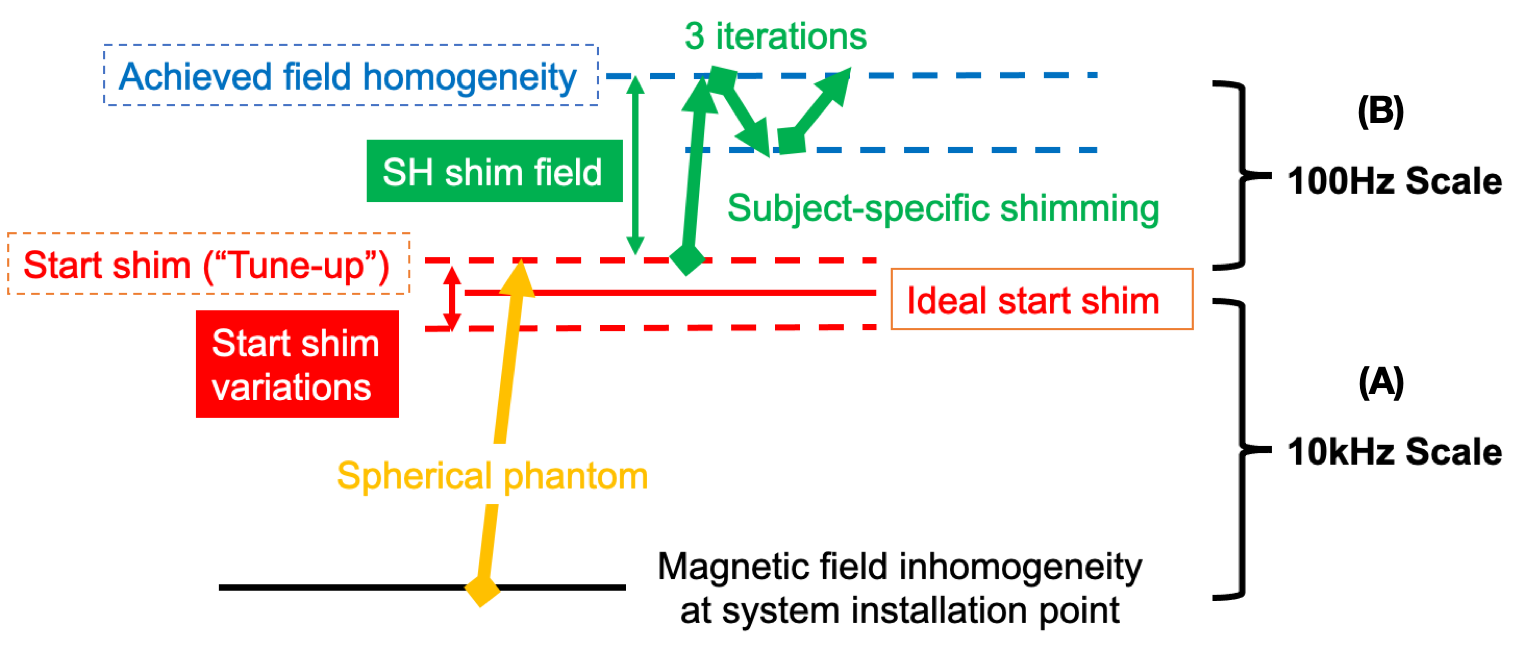


**Fig. S9** Shim method and process from the system installation point to the measuring point.

First, we will explain the ﻿terminology used for describing different shimming methods and states.

- As part of the regular maintenance, provided by the vendor, the shims are calibrated (usually up to 2^nd^ order) to optimize field homogeneity in a spherical phantom. This is the so called “Tune-up shim”. The tune-up shim optimizes field homogeneity available after the final system installation to reach a magnetic field homogeneity guaranteed by the vendor (for example, <1ppm over 40 cm DSV) (in Figure S9A). The change of gradient offsets in tune-up shim results in an absolute peak-to-peak magnetic field difference in the order of 10kHz (in Figure S10).
- Once a volunteer is positioned in the bore (as shown in Figure S9B), subject-specific shimming using the vendor-supplied shimming method will be applied. This measures the field distribution within the adjustment volume (VOI) with a dual-echo steady-state sequence, and then calculates the shim currents for improving the magnetic-field homogeneity within the VOI. The procedure usually can be performed iteratively. In practice, the start shim is the pre-defined tune-up shim settings of the vendor.
- The SH shim field simulation utilizes the difference between the tune-up shim settings and the recorded subject shim coefficient settings. However, we have found over a 2-year time period, that the tune-up shims can show differences (since maintenance is carried out approximately every 6 months, the tune-up will change). This leads to interferences between the actual tune-up shim with an ideal starting shim. Despite the small shift in gradient offsets between tune-up shims, this can cause field variations with an absolute peak-to-peak in the order of 100Hz, the same scale as a measured field map. Presumably such differences would not be at hand, if the actual tune-up shim had coincided with an ideal starting shim which is the same for each new experiment.


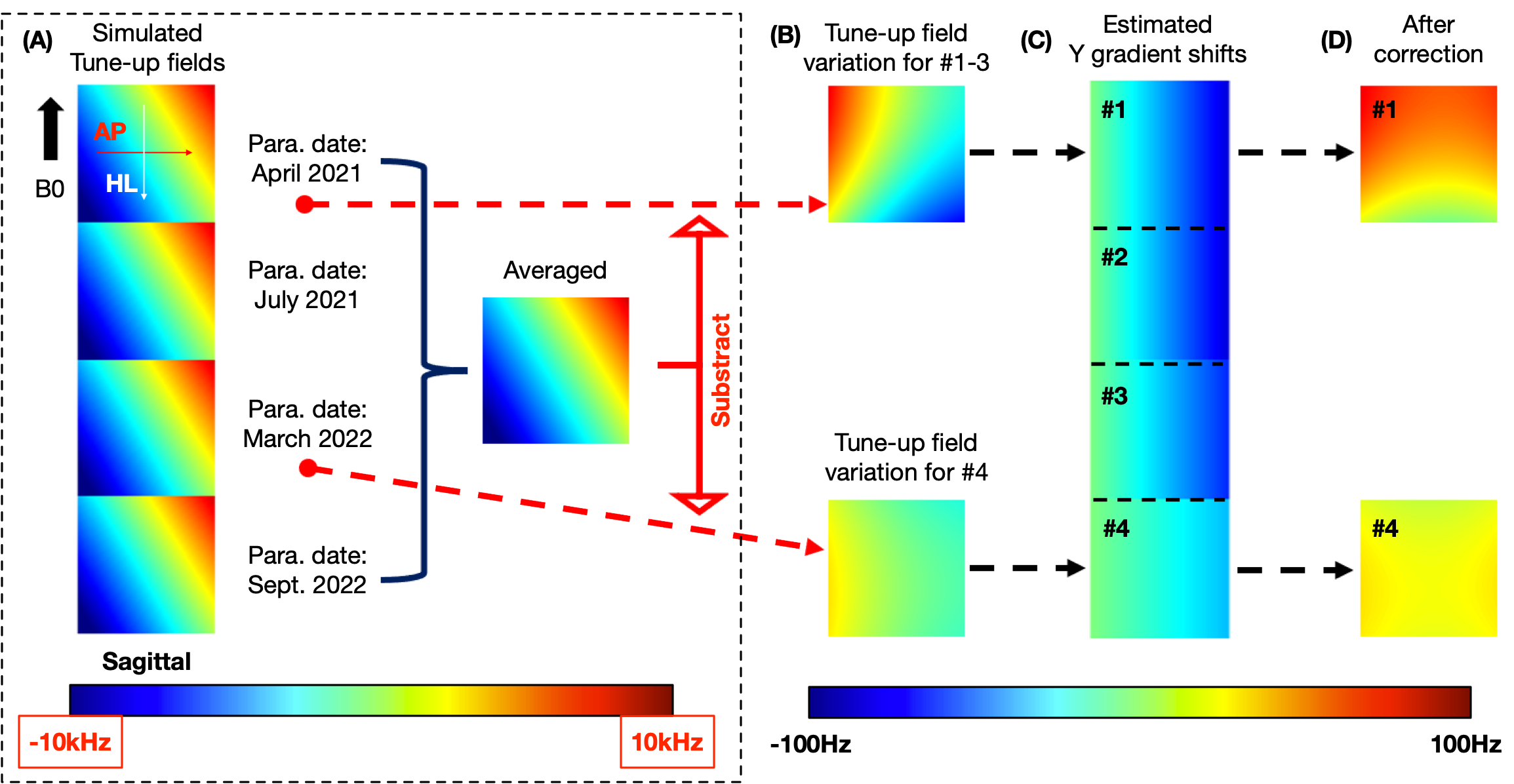


**Fig. S10** (A) Tune-up shim fields from two years period, and the ideal starting shim was assumed as an average over the obtained tune-up shim fields. The colormap is in a ±10kHz scale. (B) Estimated tune up field variation for different volunteers, the volunteers’ data were acquired on two time period as indicated by the dash read line. (C) Estimated Y gradient shifts for four volunteers, using the proposed ky maximal method. (D) Residual field after correcting the Tune-up field variation with volunteer’s estimated Y gradient shifts. Details of recorded tune-up shim coefficients and experiment shim coefficients are listed in ST2.

We examine this hypothesis by recording the tune-up shim settings over a period of two years. We found it changed 4 times during that period of time. We averaged across these four shim settings and assumed the average was the ideal starting shim (Figure S10A). For each tune-up shim the ideal start shim was subtracted to reveal the tune up shim deviation (Figure S10B). Depending on when the volunteer data was acquired, we noted that two different patterns of variation, before and after September 2022, emerged. The proposed Y gradient estimation field (Figure S10C) was able to capture the Y component shifts despite the tune-up shim variations. After the removal of this component, the corrected field (Figure S10D) shows a remanent field along the Z-axis direction. This component can later be estimated with the dipole fitting algorithm. Slight differences, such as the Y gradient estimation in in volunteer 3 compared to volunteers 1 and 2 remained. We assume this was caused by each volunteer’s physical head position and head size which also could impact the Y gradient estimation.


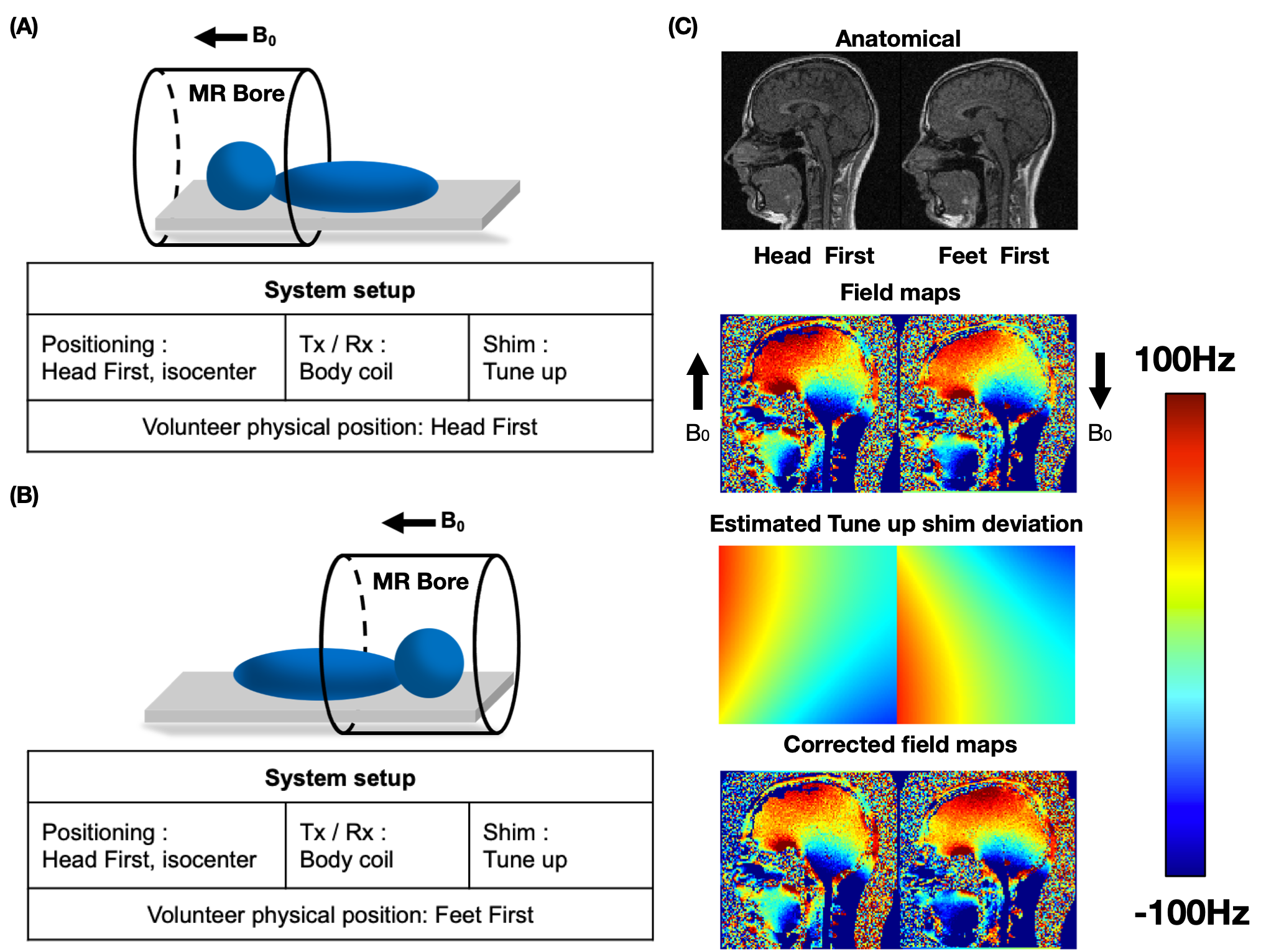


**Fig. S11** Experiment setup for head-first (A) and feet-first (B) positions. (C) First row is the anatomical images. We register the feet-first anatomical image to the head-first anatomical image using FSL FLIRT. The following field maps of feet-first position were also co-registered to the head-first position. The RMSE value (Head-first FM – Feet-first FM, within the brain mask) before and after the tune-up shim variation correction is 21.7Hz and 19.5 Hz, respectively.

An additional examination was involved in a subject measurement, where head field maps were acquired with the subject’s body laying towards (head-first position) and opposed (feet-first position) to the Z-axis direction. We use the body coil as the receiving coil (Figure S11A and S9B). The same dual-echo GRE field map sequence as described in the manuscript was applied, but with a lower resolution (2mm iso). The same field maps were expected, if the difference between the tune-up shim and ideal start shim is equal to zero within the FOV. On the contrary, the field maps are not identical. By correcting with calculated variations field, the RMSE value of the two field maps (head-first and feet-first) within the brain decreased by 10.1% (from 21.7 Hz to 19.5 Hz). Subsidiary errors in the field map are because of 1) phase unwrapping failure at low SNR region, for example, the jaw region and the brain boundaries 2) discrepancy between the assumed start shim from averaging and the ideal start shim.

**ST2**

| Tune-up shim coefficients | | | | | | | | |
| --- | --- | --- | --- | --- | --- | --- | --- | --- |
| SH terms | Z | X | Y | Z2 | ZX | ZY | X2-Y2 | XY |
| Date:  April 2022 | -704.19 | -516.47 | -1150.65 | 154.75 | -32.68 | -14.02 | -23.09 | 6.28 |
| Date:  July 2021 | -705.37 | -514.43 | -1152.20 | 157.73 | -30.55 | -15.42 | -21.67 | 4.88 |
| Date:  March 2022 | -710.86 | -513.73 | -1150.85 | 159.22 | -32.32 | -15.32 | -21.31 | 6.28 |
| Date:  Sept. 2022 | -701.06 | -513.90 | -1162.21 | 181.54 | -29.84 | -15.42 | -7.81 | 7.32 |
| Experiment shim coefficients | | | | | | | | |
| #1 | -681.39 | -516.01 | -1158.91 | 186.55 | -52.15 | -120.62 | 15.07 | 11.81 |
| #2 | -673.98 | -511.58 | -1163.49 | 220.13 | -37.62 | -100.98 | 9.31 | -2.76 |
| #3 | -679.98 | -515.46 | -1166.10 | 122.57 | -59.98 | -149.32 | -9.44 | -14.17 |
| #4 | -679.14 | -516.13 | -1145.49 | 307.37 | -34.49 | -71.33 | -97.14 | -29.43 |

**ST. 2** Recorded shim coefficients for tune-up and experiment. The experiment shim data for volunteer 1 to 3 are acquired using the tune-up shim setting as shown in gray, where for volunteer 4 using the tune-up shim setting as shown in yellow.
